# Lasmiditan and 5-Hydroxytryptamine in the rat trigeminal system; expression, release and interactions with 5-HT_1_ receptors

**DOI:** 10.1186/s10194-022-01394-z

**Published:** 2022-02-17

**Authors:** Jacob C. A. Edvinsson, Aida Maddahi, Isabella M. Christiansen, Philip V. Reducha, Karin Warfvinge, Majid Sheykhzade, Lars Edvinsson, Kristian A. Haanes

**Affiliations:** 1 Department of Clinical Experimental Research, Glostrup Research Institute, Rigshospitalet Glostrup, 2600 Glostrup, Denmark; 2 Department of Drug Design and Pharmacology, Faculty of Health and Medical Sciences, University of Copenhagen, Copenhagen, Denmark; 3 Division of Experimental Vascular Research, Department of Clinical Sciences, Lund University Hospital, Lund, Sweden; 4 Department of Biology, University of Copenhagen, Copenhagen, Denmark

**Keywords:** 5-HT, Lasmiditan, CGRP, Migraine, Trigeminal system

## Abstract

**Background:**

5-Hydroxytryptamine (5-HT) receptors 1B, 1D and 1F have key roles in migraine pharmacotherapy. Selective agonists targeting these receptors, such as triptans and ditans, are effective in aborting acute migraine attacks and inhibit the in vivo release of calcitonin gene-related peptide (CGRP) in human and animal models. The study aimed to examine the localization, genetic expression and functional aspects of 5- HT_1B/1D/1F_ receptors in the trigeminal system in order to further understand the molecular sites of action of triptans (5-HT_1B/1D_) and ditans (5-HT_1F_).

**Methods:**

Utilizing immunohistochemistry, the localization of 5-HT and of 5-HT_1B/1D/1F_ receptors was examined in rat trigeminal ganglion (TG) and combined with quantitative polymerase chain reaction to quantify the level of expression for 5-HT_1B/1D/1F_ receptors in the TG. The functional role of these receptors was examined *ex vivo* with a capsaicin/potassium induced 5-HT and CGRP release.

**Results:**

5-HT immunoreactivity (ir) was observed in a minority of CGRP negative C-fibres, most neuron somas and faintly in A-fibres and Schwann cell neurolemma. 5-HT_1B/1D_ receptors were expressed in the TG, while the 5-HT_1F_ receptor displayed a weak ir. The 5-HT_1D_ receptor co-localized with receptor activity-modifying protein 1 (RAMP1) in Aδ-fibres in the TG, while 5-HT_1B_-ir was weakly expressed and 5-HT_1F_-ir was not detected in these fibres. None of the 5-HT_1_ receptors co-localized with CGRP-ir in C-fibres.

5-HT_1D_ receptor mRNA was the most prominently expressed, followed by the 5-HT_1B_ receptor and lastly the 5-HT_1F_ receptor. The 5-HT_1B_ and 5-HT_1D_ receptor antagonist, GR127935, could reverse the inhibitory effect of Lasmiditan (a selective 5-HT_1F_ receptor agonist) on CGRP release in the soma-rich TG but not in soma-poor TG or dura mater. 5-HT release in the soma-rich TG, and 5-HT content in the baseline samples, negatively correlated with CGRP levels, showing for the first time a physiological role for 5-HT induced inhibition.

**Conclusion:**

This study reveals the presence of a subgroup of C-fibres that store 5-HT. The data shows high expression of 5-HT_1B/1D_ receptors and suggests that the 5-HT_1F_ receptor is a relatively unlikely target in the rat TG. Furthermore, Lasmiditan works as a partial agonist on 5-HT_1B/1D_ receptors in clinically relevant dose regiments.

**Supplementary Information:**

The online version contains supplementary material available at 10.1186/s10194-022-01394-z.

## Introduction

Migraine headache has historically been considered a vascular disorder where vasodilatation of cranial (extracerebral) blood vessels generates activation of trigeminal afferents which is associated with pain [1]. This view has over time been challenged by the hypothesis that favour a neurological origin [2]. The generation of a migraine attack has been proposed to be initiated in the central nervous system (CNS), involving regions such as the hypothalamus and brainstem, much of this is associated with the premonitory symptoms found in many patients [3]. In support, neuroimaging studies on migraineurs with daily scanning over 30 days have revealed hypothalamic activation in the premonitory phase and increased activity in the ictal phase for brainstem regions (e.g. pontine tegmentum) [4, 5].

Although the origin of migraine may be in the CNS, the perceived pain is referred through the trigeminovascular system (TVS), which includes the trigeminal ganglion (TG), and bridge the trigeminocervical complex with the brain [6]. Due to the low blood-brain barrier (BBB) permeability and clinically proven effectiveness of triptans and calcitonin gene-related peptide (CGRP) monoclonal antibodies, the TG and its afferents are a likely target in migraine pharmacotherapy.

Triptans are clinically well established and effective drugs indicated for the treatment of acute migraine headache attacks [7]. The available triptans are functionally homogenous in their mode of action with differences to be found in bioavailability and pharmacokinetics (e.g. half-life) [8, 9]. These drugs were originally developed on the assumption that reduced vasodilatation would alleviate migraine headache, however, we now know that 5-HT_1_ receptor agonists (e.g. sumatriptan) can substantially inhibit release of neuropeptides such as CGRP and Substance P (SP) [10, 11], thus revealing a potential mechanism of action relevant to migraine therapy [12].

Triptans are 5-hydroxytryptamine (5-HT) receptor agonists with high affinity for the 5-HT_1B/1D_ receptor subtypes and with moderate affinity for 5-HT_1A_ and 5-HT_1F_ receptors [13, 14]. They elicit potent vasoconstrictive effects in cranial arteries mainly via the 5-HT_1B_ receptor. The 5-HT_1B_ receptor is expressed on smooth muscle cells in cerebral [15], meningeal [16] and coronary arteries [17]. While the 5-HT_1D_ receptor is less effective in inducing vasoconstriction [18], it is prominently expressed in the TG [19, 20]. A 5-HT_1D_ agonist was found to block both peripheral and central aspects of TVS activation [21]. Lasmiditan, a recently developed selective 5-HT_1F_ receptor agonist [22], does not induce vasoconstriction but has both a CGRP release inhibiting potential and an anti-migraine effect [23–25].

Pharmacological studies have suggested that 5-HT_1D_ receptor agonists should inhibit trigeminal nociceptive traffic though less potently than via the 5-HT_1B_ receptor [26]. The same study concluded that blocking the 5-HT_1D_ receptor with the selective antagonist BRL-15572 reduced the inhibitory effect of several triptans, thus showing that 5-HT_1D_ receptors may yet have a role to play in migraine pharmacotherapy. It should be noted that the only attempted clinical trial with a specific 5-HT_1D_ receptor agonist (PNU-142633) proved ineffective in aborting migraine attacks [27]. However, the results of the study has been deemed inconclusive [14] in part due to low sample size and uncertain receptor affinity [28].

While 5-HT is broadly reported to be expressed in mast cells and platelets, the inconclusive expression of 5-HT in nerve fibres innervating cerebral arteries have been suggested to be attributed to extracellular uptake [29]. Tryptophan hydroxylase (TPH), the rate-limiting enzyme in the 5-HT biosynthesis, immunoreactivity (ir) has been found in both thin varicose nerve fibres (likely C-fibres) and thicker nerve bundles (likely Aδ-fibres) inhabiting the rat dura mater [29]. However, the presence of TPH was not deemed sufficient in synthesizing measurable amounts of 5-HT in these fibres, making it an uncertain marker for “authentic” serotonergic innervation [29]. A previous study, utilizing real-time polymerase chain reaction, also faced technical difficulties in measuring TPH mRNA levels in central serotonergic neurons due to its low levels [30].

The aim of this study was to revisit the field of 5-HT_1_ receptor subtypes to shed light on their probable site of action within the trigeminal nerve. In addition, we wanted to study the expression of their natural ligand, 5-HT, within this region. To achieve this, we applied immunohistochemistry (IHC) to obtain a detailed visualization of 5-HT and its relevant receptors. The expression of 5-HT_1_ receptor subtypes was compared utilizing quantitative polymerase chain reaction (qPCR) and a light emission measurement. Lastly, functional aspects of CGRP and 5-HT release were explored in *ex vivo* TG and a hemi-skull model.

## Materials & methods

### Animals

All animal procedures were performed in accordance with the European Community Council Directive on ‘The Protection of Animals Used for Scientific Purposes’ (2010/63/EU). Animal procedures were approved by the Lund University Animal Ethics Committee (M43–07) and The Danish Animal Experimentation Inspectorate respectively.

Adult male Sprague Dawley rats (260-300 g), housed in groups of 2-3 rats together in Tall IVC Rat Cages (Innovive), were utilized for the IHC part of this study. For CGRP and 5-HT release experiments additional Sprague Dawley rats (260-300 g) were used. These rats were housed in Eurostandard cages (Type VI with 123-Lid) in groups of 2-6 rats together.

All animals were kept under standard laboratory conditions (humidity-controlled, and 12/12h light-dark cycle, with dark beginning at 7 p.m.) with access to chow (RM1, SDS) and water *ad libitum*. All animals were first anaesthetized by CO_2_ inhalation and subsequently decapitated prior to experiments.

### RNA isolation and qPCR

TGs from 8 male rats were carefully dissected and immediately frozen on liquid nitrogen for RNA extraction. RNA was extracted from all samples using RNeasy® Plus Mini kit (Qiagen, Hilden, Germany) in accordance to the manufacturer’s protocol. Total RNA concentration was determined using a GeneQuant Pro spectrophotometer (Amersham Pharmacia Biotech, Uppsala, Sweden). A ratio of sample absorbance at 260/280 nm in the range of 1.8 to 2.0 was deemed acceptable. 1 μg total RNA was used in a 20 μL reverse transcript reaction using SuperScript® III First-Strand Synthesis Super Mix (Invitrogen, Carlsbad, CA, USA) for qPCR. In order to detect genomic DNA, a reverse transcription negative control was performed simultaneously for each sample, but in the absence of SuperScript III Reverse Transcriptase, for each sample to detect genomic DNA. The obtained cDNA was diluted to a total volume of 80 μL and stored frozen at -20 °C. Primer sequences (for details, see Table 1) were specific for the genes of interest and were designed using Primer Express 3.0 software (PE Applied Biosystems, Foster city, CA, USA) and synthesized by TAG Copenhagen A/S (Copenhagen, Denmark). Glyceraldehyde-3-phosphate dehydrogenase (GAPDH) was utilized as a housekeeping gene to which the gene expressions were normalized against.

**Table 1:** Details of qPCR primer sequences

Primer	Forward	Reverse
5-HT_1B_	5`-TCCGGGTCTCCTGTGTACGT -3`	5`-GGCGTCTGAGACTCGCACTT -3`
5-HT_1D_	5`-GCATCTCTGTGTCATCGCTCT -3`	5´-ATGTGTTCACCAGGCAGTCA -3`
5-HT_1F_	5`-GAACGCAAAGCAGCCACTAC -3`	5`-AGGTAACCAAGCCATGCCAA -3`
GAPDH	5`-CTGCACCACCAACTGCTTAGG -3`	5´-TCAGCTCTGGGATGACCTTGC- 3`

The qPCR was performed in 20 μL total volume reaction consisting of 2 μL diluted cDNA, 0.5 μM of each primer, and 10 μL Fast SYBR™ Green Master Mix (Applied Biosystems, CA, USA), and 7 μL RNAase free water in a Step One Plus Real Time PCR System (Applied Biosystems, CA, USA). The qPCR reaction had the following thermal profile: Holding stage at +50 °C for 2 min and +95 °C for 10 min, followed by 40 PCR cycles at +95 °C for 15 s and +60 °C for 1 min. Each sample was examined in duplicate and a blank control (without template) was used in all experiments. After amplification a melting curve analysis was performed to verify that each primer pair generated only one PCR product of expected size.

### Immunohistochemistry

TGs were carefully dissected and incubated in 4 % paraformaldehyde in phosphate buffered saline (PBS) for 2-4 h at room temperature. The fixated TGs were immersed in 10 % sucrose (Sigma) in Sorensen’s phosphate buffer (2-4 h, +4 °C) and subsequently in a 25 % solution overnight. The following day, the TGs were embedded in a gelatine medium (30 % egg albumin, 3 % gelatine) and cryosectioned at 10 μm. The sections were mounted on microscope slides (Superfrost, ThermoFisher) and stored at -20 °C until use.

The TG sections were rehydrated and permeabilized in 0.25 % Triton X-100 diluted in PBS (PBS-T; Sigma) for 2×15 minutes. Primary antibodies (for details, see Table 2) diluted in PBS-T containing 1 % bovine serum albumin (BSA; Sigma) were applied to the sections which were incubated at +4 °C overnight. Sections were subsequently rinsed of excess antibodies in PBS-T for 2×15 min and incubated with secondary antibodies (for details, see Table 2), diluted in PBS-T, for 1 h in a dark room. Afterwards, excess secondary antibodies were rinsed with PBS-T 2×15 min and cover glass was mounted with anti-fading medium Vectashield containing 4',6-diamidino-2-phenylindole (DAPI; Vector Laboratories, Burlingame CA, USA). For double immunohistochemical stainings, the process was repeated on the same sample with an additional primary and secondary antibody. Negative controls were simultaneously made following the same procedure but in the absence of primary antibodies. The mounted sections were examined with an epifluorescence microscope (Nikon 80i, Tokyo, Japan) coupled to a Nikon DS-2 MV camera. Images were obtained with NIS basic research software (Nikon, Japan) and processed into figures using Adobe Photoshop CC 2020 (Adobe Systems, Mountain View, CA, USA).

**Table 2:** Details of primary and secondary antibodies

**Primary antibodies**				
Antigen	Dilution	Species	Immunogen	Supplier
CGRP (ab81887)	1;100	Mouse	Rat alpha-CGRP	Abcam, Cambridge, UK
5-HT_1B_ (ab13896)	1;100	Rabbit	Amino acids 8–26 and 263–278 of 5- HT1B	Abcam, Cambridge, UK
5-HT_1D_ (ab13895)	1;100	Rabbit	Amino acids 1–18 and 251–267 of rat 5-HT1D	Abcam, Cambridge, UK
5-HT_1F_ (SP4043P)	1;100	Rabbit	N-terminus extracellular domain of human 5-HT1F	Acris Antibodies, San Diego, CA, USA
5-HT (20079)	1;100	Goat	Serotonin whole molecule conjugated to BSA with paraformaldehyde.	Immunostar, Hudson, WI, USA
5-HT_1F_ (TA340657)	1;100	Rabbit	5-HT_1F_ Receptor antibody was raised against synthetic 19 amino acid peptide from N-terminal extracellular domain of human 5HT_1F_ Receptor	Origene, Rockville, MD, USA
CASPR (MABN69)	1;100	Mouse	Recombinant protein corresponding to rat contactin associated protein 1.	EMD milipore Corp. Temecula, CA, USA
RAMP1 (844)	1;200	Goat	C-terminal of human RAMP1	Merck & Co, Inc., West Point, PA, USA.
MBP (MA5-15922)	1;100	Mouse	Purified recombinant fragment of human MBP expressed in E.Coli.	Thermo Fisher Scientific, Waltham, MA, USA.
**Secondary antibodies**				
Product	Dilution	Immunogen	Supplier	
FITC	1;100	Anti-rabbit	Cayman Chemical, Ann Arbor, MI, USA	
Alexa flour 594	1;100	Anti-mouse	Jackson Immunoresearch Laboratories, Inc., West Grove, PA, USA	
Dyelight 488	1;100	Anti-rabbit	Jackson Immunoresearch Laboratories, Inc., West Grove, PA, USA	
Cy3	1;200	Anti-goat	Jackson Immunoresearch Laboratories, Inc., West Grove, PA, USA	
FITC	1;100	Anti-mouse	Jackson Immunoresearch Laboratories, Inc., West Grove, PA, USA	

### Light intensity measurements

The level of ir was compared between 5-HT_1B/D/F_ receptors using microphotographs from IHC results. To reduce bias and confounding factors, all measured areas were compared (Fig. 5) and measurements were only made for primary antibodies from the same species (rabbit) utilizing the same secondary antibody.

For 5-HT_1_ receptors, each TG was microphotographed (18 per antibody, *n* = 6). All microphotographs were taken in greyscale at 333 ms exposure in the FITC filter and each primary antibody (all rabbit, 1:100) had been labelled with the same secondary antibody (FITC, anti-rabbit, 1:100).

All microphotographs were taken in areas of 0.09375 mm^2^ containing at least 10 visible neurons. The images were converted to greyscale, neurons were outlined using a freehand tool in the ImageJ software [31]. All light emission measurements were performed in ImageJ and the data was processed in GraphPad Prism 8.0.

### CGRP and 5-HT release

Each TG was carefully dissected and transferred to an Eppendorf tube containing 1 mL synthetic interstitial fluid (SIF, composition: 108 mM NaCl, 3.5 mM KCl, 3.5 mM MgSO_4_, 26 mM NaHCO_3_, 11.7 mM NaH_2_PO_4_, 1.5 mM CaCl_2_, 9.6 mM Sodium Gluconate, 5.6 mM glucose and 7.6 mM sucrose; pH 7.4.) which was incubated at +37°C for 30 min.

To reduce the baseline signals, the tissues were washed five consecutive times in 300 μL SIF at +37°C for 10 min. Subsequently, 200 μL samples from each tube was collected to assess a baseline for each tissue. CGRP and 5-HT release was induced by immersion in 60 mM K^+^-SIF, where an equimolar amount of Na^+^ was replaced by K^+^ to maintain osmolality or 100 nM capsaicin, for 10 minutes after which 200 μL samples were collected. 50 μL enzyme immunoassay buffer was added to each sample which was then stored frozen at -20°C until analysis. For the experiments with agonist/antagonists, the experiments were performed sequentially, with pre-incubation for 10 minutes for each [32]. For Lasmiditan (ACHEMblock, L22337, USA) a 30 mM stock in dimethyl sulfoxide (DMSO) was prepared, GR127935 (Tocris, UK) was dissolved in water and kept frozen as stocks of 3 mM. BRL15572 (Tocris, UK) was dissolved in DMSO and kept frozen as stocks of 1.25 mM. All experiments were performed with matching vehicles.

The samples were processed using commercial enzyme immunoassay kits, Human CGRP enzyme-linked immunosorbent assay (ELISA) KIT (SPIbio, Paris, France) for CGRP and Serotonin ELISA kit, (Enzo Life sciences, ADI-900-175, detection limit 0.293 ng/ml). The protocol was performed according to the manufacturer’s instructions and the optical density was measured at 410 nm for both using a micro-plate photometer (Tecan, Infinite M200, software SW Magellan v.6.3, Männedorf, Switzerland).

### Statistics

Quantitative data obtained from release experiments was analysed using GraphPad Prism 8.0, and presented as mean ± SEM. The differences between the variables were compared with a two-sided paired Student’s t-test if the data passed the Shapiro-Wilks test for normality. For the correlation, the data is presented as individual points with p-values and the R^2^-value was obtained from the correlation analysis in GraphPad 8.0.

Data from the real time PCR was analysed with the comparative cycle threshold (C_t_) method [33]. The C_t_ values for GAPDH, 5-HT_1B_, 5-HT_1D_ and 5-HT_1F_ mRNA were used as a reference to quantify the relative amount of mRNA. The relative amount of mRNA was calculated using the formula X_0_/R_0_ = 2^(CtR-CtX)^. Where X_0_ is the amount of target mRNA, R_0_ is the amount of housekeeping gene mRNA, C_t_R is the C_t_ value of the housekeeping gene and C_t_X is the C_t_ value of the target. Data is expressed as mean ± SEM and n refers to the number of rats. Statistical analyses for the real time PCR and the light emission measurement were performed with Kruskal Wallis non-parametric test with Dunn`s post-hoc test, using GraphPad Prism 8. *P<0.05, **P<0.01 and ***P<0.001 were considered significant.

## Results

### 5-HT_1_ receptor light emission measurements

**Fig. 1:**
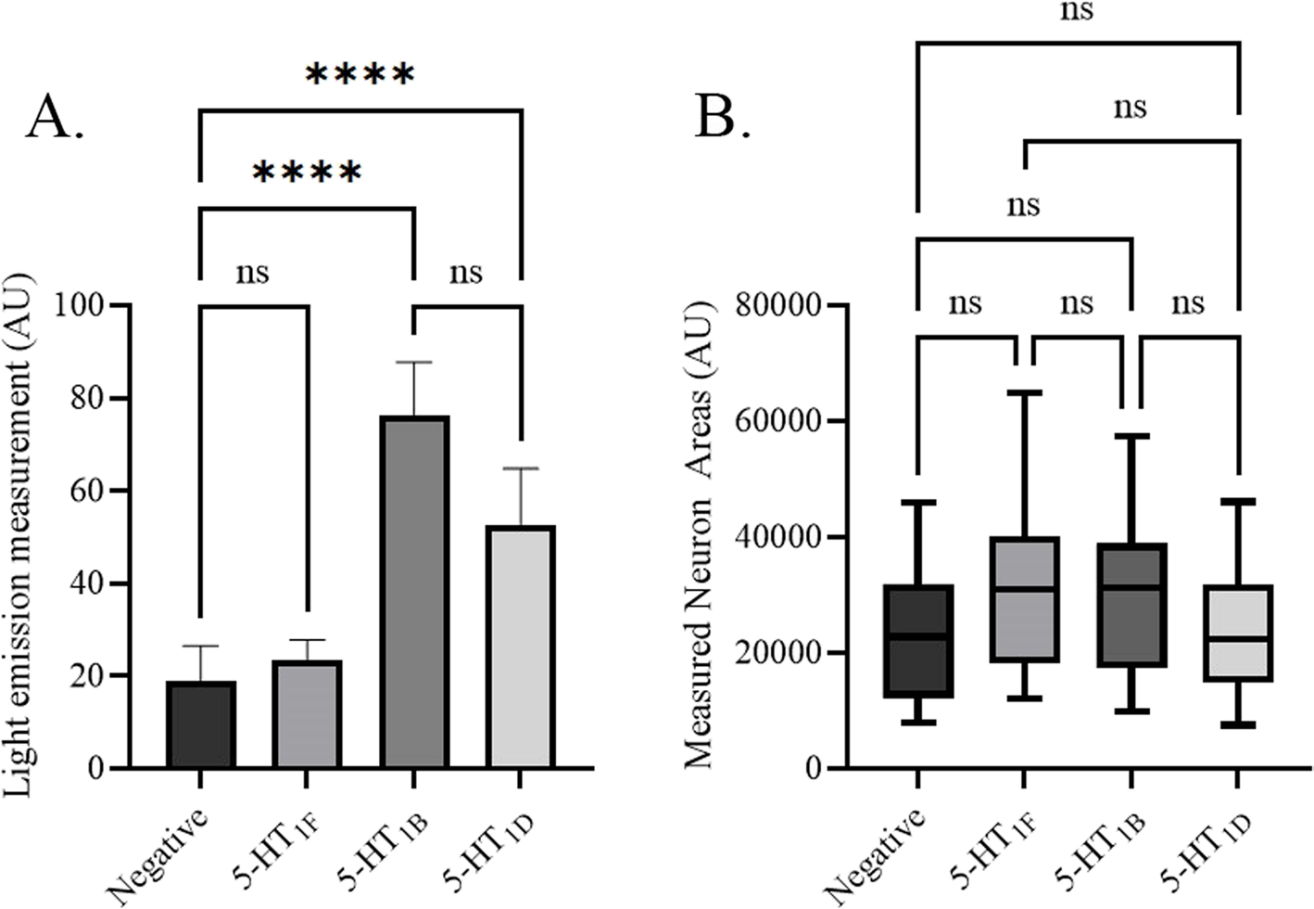
5-HT_1_ receptor light emission measurement, from representative areas of 0.09375 mm^2^ in each slide (18 microphotographs for each antibody, *n* = 6). All microphotographs were taken in greyscale at 333 ms exposure in FITC filter. Each primary antibody (all rabbit, 1:100) was labelled with the same secondary antibody (FITC, anti-rabbit, 1:100). Measurements were performed in imageJ using the freehand tool to trace neuron areas. All data presented as mean ± 1 standard deviation. The data was further analysed with Kruskal Wallis non-parametric test with Dunn`s post-hoc test. **A** 5-HT_1B_ (76.4 ± 11.1 AU) and 5-HT_1D_ (52.8 ± 11.6 AU) were the most prominently emitted. 5-HT_1F_ (23.6 ± 4.2 AU) did not significantly differ from the negative control (19.1 ± 7.2 AU). **B** No significant difference between the measured neuron areas was found

Light emission measurements of IHC stained TG neurons demonstrated that 5-HT_1F_ (23.6 ± 4.2 AU, n = 6) was too weakly expressed in the trigeminal neuron cell bodies to significantly differentiate from the negative control (19.1 ± 7.2 AU, *n* = 6; *p* < 0.05) (Fig. 1A). In addition, measurements for 5-HT_1B_ (76.4 ± 11.1 AU, *n* = 6) and 5-HT_1D_ (52.8 ± 11.6 AU, *n* = 6) receptors significantly differentiated from the negative control (*p* > 0.05) but not from each other (n.s). No significant difference in the measured areas was detected (Fig. 1B).

### Immunohistochemistry

**Fig. 2:**
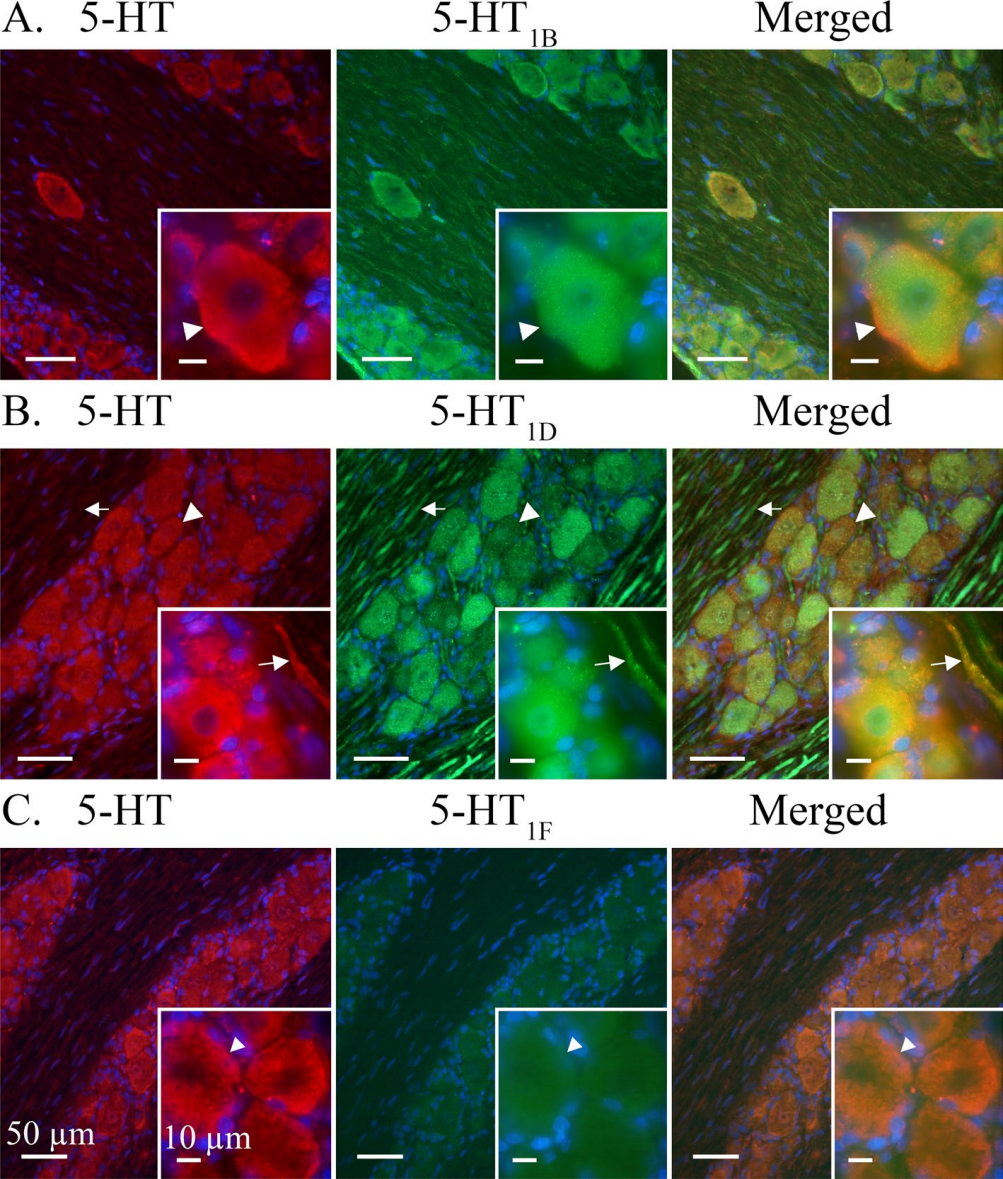
Expression of 5-HT in relation to 5-HT_1B/1D/1F_ receptors. A majority of observed TG neurons expressed 5-HT-ir in a heterogeneous granular pattern. This pattern was reminiscent of cytoplasmic vesicles and which was often visualized close to the cell membrane. 5-HT was generally weakly expressed in Aδ-fibres, Schwann cells and satellite glial cells. **A** 5-HT_1B_ receptor ir was found in all observed neuronal cell bodies and more faintly in Aδ-fibres. 5-HT_1B_-ir was observed in an evenly distributed granular pattern across the neuron cytoplasm. 5-HT_1B_ co-localized with 5-HT in the neuronal cytoplasm. Insert: Arrowheads mark a neuron where 5-HT is intensely expressed close to the cell membrane and 5-HT_1B_ is more evenly distributed across the whole cell body. **B** A majority of observed neurons displayed 5-HT_1D_ receptor ir in a vesicular pattern. Unlike the intense and evenly distributed ir of 5-HT_1B_ receptor, the 5-HT_1D_ receptor displayed both strong and weak ir in different subsets of neurons. Arrowheads mark a neuron with a fainter 5-HT_1D_ expression than a neighbouring neuron. Furthermore, 5-HT_1D_ displayed a distinct Aδ-fibre ir. Arrows mark a visible Aδ-fibre. Double IHC showed that 5-HT_1D_ co-localized with 5-HT in the neuron cell bodies. 5-HT expression could be observed in 5-HT_1D_-positive Aδ-fibres, albeit inconsistent and faint. Insert: Arrows mark Aδ-fibre expressing both 5-HT_1D_ and 5-HT ir. **C** 5-HT co-localized with the faintly positive 5-HT_1F_ receptor in neuron cell bodies. No nerve fibre ir was observed for the 5-HT_1F_ receptor. Insert: Arrowhead marks a 5-HT positive neuron with a faint 5-HT_1F_ expression

**Fig. 3:**
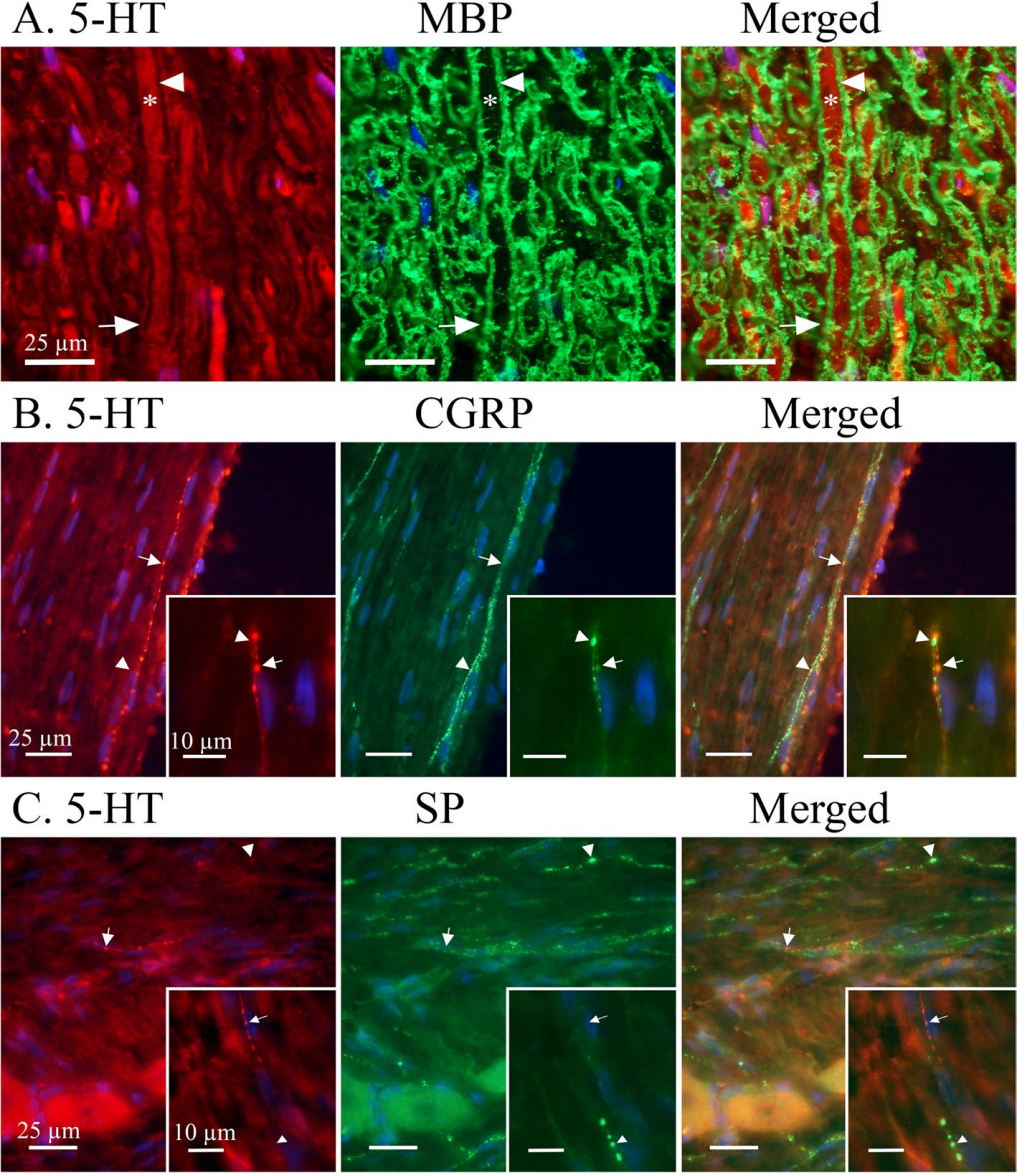
Expression of 5-HT in relation to myelin basic protein (MBP), SP and CGRP. **A** MBP showed a distinct staining of myelin sheath flanking cross-sectioned Aδ-fibre axons. A robust MBP-ir was observed (Arrowhead) between the Aδ-fibre axon and Schwann cell neurolemma. An irregular, and sometimes faint, expression of 5-HT could be observed in Aδ-fibre axons (Asterix) and just outside the myelin sheath (Arrow), indicative for the neurolemma of the Schwann cell containing its cytoplasm. **B** A minority of observed C-fibres expressed 5-HT-ir, these were more frequently found in proximity to Redlich-Obersteiner’s zone. CGRP-ir was more abundantly expressed in C-fibres and did not co-localize with 5-HT. Arrows mark 5-HT positive C-fibre boutons and arrowheads mark CGRP positive C-fibre boutons. Insert: Two thin C-fibres entwining and differentially expressing either CGRP or 5-HT. **C** Similarly, SP could be found concentrated to C-fibre boutons (Arrowhead) throughout the TG. The few 5-HT positive C-fibres (Arrow) were not found to co-localize with SP positive C-fibres. Insert: A thin C-fibre expressing 5-HT-ir (Arrow) above a seemingly larger SP positive C-fibre (Arrowhead)

**5-HT**-ir was found in the cytoplasm of all observed neuronal cell bodies where it co-localized with 5-HT_1B/1D/1F_ receptor ir (Fig. 2A-C). The 5-HT-ir was displayed in a granular pattern which often concentrated close to the cell membrane. 5-HT-ir was sporadically and faintly expressed in Schwann cells and satellite glial cells (SGCs). In addition, 5-HT-ir could be unevenly observed in Aδ-fibres and Schwann cell neurolemma but did not co-localize with myelin basic protein (MBP) (Fig. 3).

When double stained with CGRP and SP, 5-HT-ir could be detected in CGRP or SP positive neuron cell bodies but did not co-localize in CGRP or SP immunoreactive C-fibres. Instead, 5-HT-ir was observed in a subset of C-fibres (Fig. 3B-C) fewer than those expressing CGRP or SP and were mainly observed proximal to the Redlich-Obersteiner’s zone (i.e., the root entry zone). In addition, 5-HT-ir was mainly observed in very thin (<0.5 μm in diameter) C-fibre axons, while CGRP-ir was observed in all C-fibre axon ranges (0.2 μm – 1.5 μm in diameter).

**Fig. 4:**
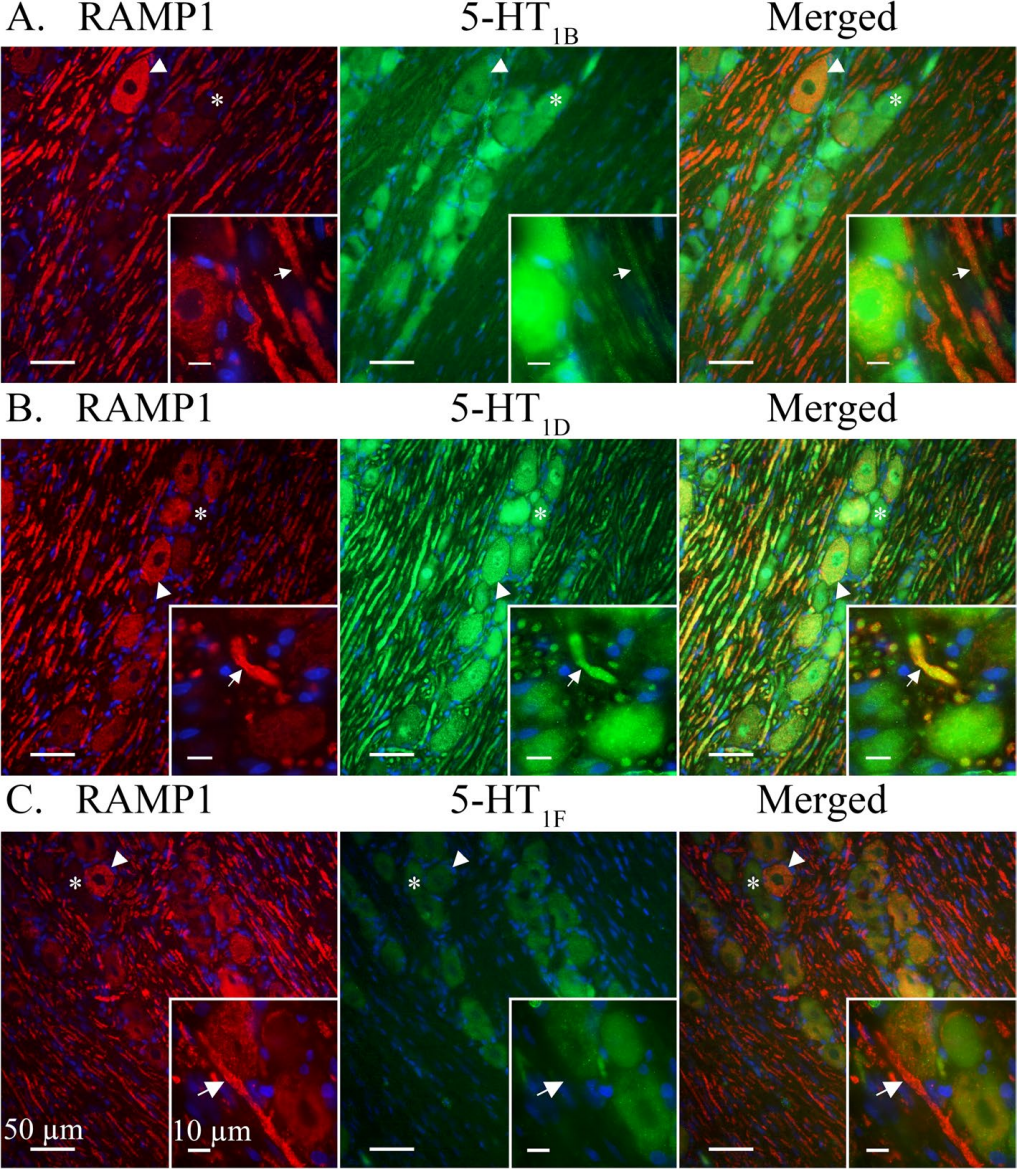
Double staining CGRP receptor element RAMP1 and 5-HT_1B/D/F_ receptors. RAMP1 was expressed in larger neurons cell bodies (Arrowheads) and Aδ-fibres (Arrows). Several 5-HT_1_ receptor positive neurons did not display RAMP1-ir (Asterixes). **A** 5-HT_1B_ receptor ir co-localized with RAMP1 in larger neurons (Arrowhead). A weak ir for the 5-HT_1B_ receptor could be detected in the RAMP1 labelled Aδ-fibres (Arrows). **B** 5-HT_1D_ receptor ir co-localized with RAMP1 in Aδ-fibres (Arrow) and larger neurons (Arrowhead). **C** 5-HT_1F_ receptors were not expressed in observed RAMP1 positive Aδ-fibres (Arrow). Weakly positive 5-HT_1F_ neurons could be observed in neuron cell bodies positive to RAMP1 (Arrowhead)

**Fig. 5:**
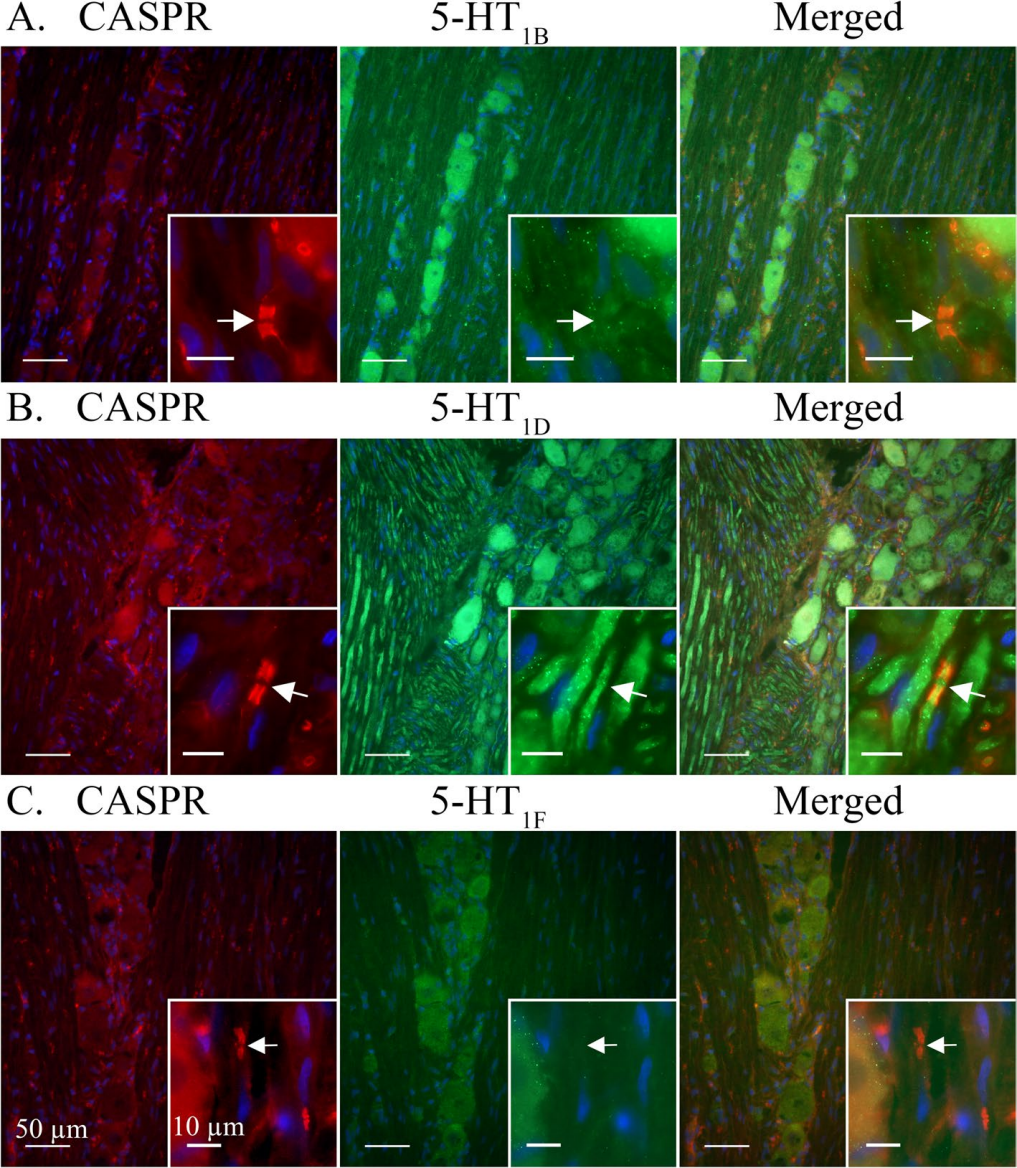
5-HT_1B/D/F_ receptors co-staining with the paranodal marker CASPR. Arrows mark nodes of Ranvier in CASPR labelled Aδ-fibres. **A** Ir for 5-HT_1B_ receptors was observed in neuron cell bodies and faintly in CASPR positive Aδ-fibres. **B** 5-HT_1D_ receptors were clearly expressed in CASPR positive Aδ-fibres and neuron cell bodies. **C** A weak ir for 5-HT_1F_ receptors was detected in some neuron cell bodies while no ir was found in Aδ-fibres

**Fig. 6:**
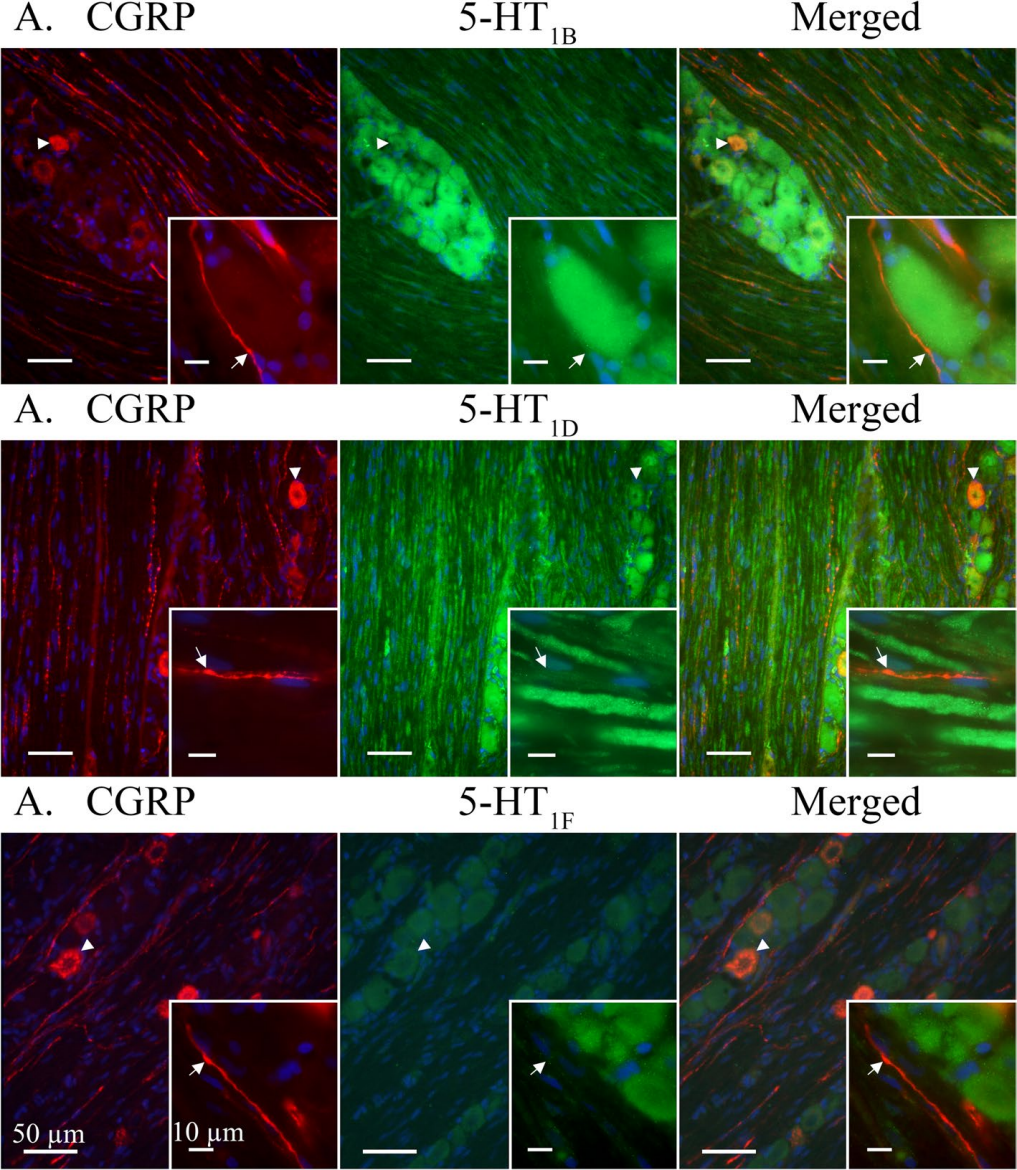
5-HT_1B/D/F_ receptors co-staining with CGRP. CGRP-ir revealed a vesicular staining pattern in neuron cell bodies and C-fibres. **A** CGRP co-localized with 5-HT_1B_ receptors in some neuron cell bodies (Arrowhead). No expression of the 5-HT_1B_ receptor was observed in CGRP positive C-fibres. Insert: CGRP immunoreactive C-fibre adjacent to 5-HT_1B_ positive neuron cell body. Arrow marks a visible C-fibre bouton. **B** CGRP-ir co-localized with 5-HT_1D_ in neuron cell bodies (Arrowhead). No expression of the 5-HT_1D_ receptor was detected in the CGRP immunoreactive C-fibres. Insert: CGRP positive C-fibre intermingled between 5-HT_1D_ positive Aδ-fibres. Arrow marks a visible C-fibre bouton. **C** CGRP co-localized with weakly positive 5-HT_1F_ receptors in neuron somas. No expression of the 5-HT_1F_ receptor was detected in C-fibres. Insert: CGRP positive C-fibre in proximity to 5-HT_1F_ positive neuron cell bodies. Arrow marks a visible C-fibre bouton

**5-HT**_**1B**_-ir was detected in most observed trigeminal neuron somas. 5-HT_1B_-ir often presented in an evenly distributed granular pattern, reminiscent of cytoplasmic vesicles, across the neuron cytoplasm but could sometimes be seen concentrated close to the cell membrane (Fig. 2A). In addition, 5-HT_1B_-ir could be faintly observed in Schwann cell neurolemma and irregularly in SGCs. Although all observed neuron cell bodies were positive for 5-HT_1B_ only a faint ir could be observed in Aδ-fibres positive for receptor activity-modifying protein 1 (RAMP1) (Fig. 4A) or contactin associated protein 1 (CASPR) (Fig. 5A). Similarly, 5-HT_1B_-ir was detected in CGRP positive neurons but 5-HT_1B_-ir was not observed in CGRP positive C-fibres (Fig 6A).

5-HT_1D_-ir was detected in a majority of observed trigeminal neuron somas. 5-HT_1D_-ir was observed as a robust granular staining across the neuron or a more vesicular ir with a weaker cytoplasmic staining. In both cases, 5-HT_1D_-ir in or around the nuclei could be observed (Fig. 2B). Furthermore, 5-HT_1D_-ir was at times faintly observed in SGCs but was not seen in Schwann cells. In contrast to 5-HT_1B_-ir, 5-HT_1D_-ir was prominently expressed in Aδ-fibres where it could be seen to co-localize with RAMP1 (Fig. 4B). Though expressed in the same Aδ-fibre, 5-HT_1D_-ir was not observed to co-localize with the integral membrane protein CASPR (Fig. 5B). In agreement with 5-HT_1B_, 5-HT_1D_-ir was expressed in CGRP positive neuron cell bodies but was not observed in CGRP positive C-fibres (Fig. 6B).

5-HT_1F_-ir was faintly detected in all observed trigeminal neuron somas. Due to the weak expression, no clear compartmentalization within the cytoplasm could be distinguished (Fig. 2C). No ir for 5-HT_1F_ was observed in SGCs or Schwann cells. Similarly, 5-HT_1F_ was not observed in Aδ-fibres, marked with RAMP1 (Fig. 4C) and CASPR (Fig. 5C), or C-fibres, marked with CGRP (Fig. 6C).

### 5-HT_1B_, 5-HT_1D_ and 5-HT_1F_ receptor mRNA expression in rat TG

Levels of mRNA expression for 5-HT_1B/1D/1F_ receptors were measured with qPCR.

**Fig. 7:**
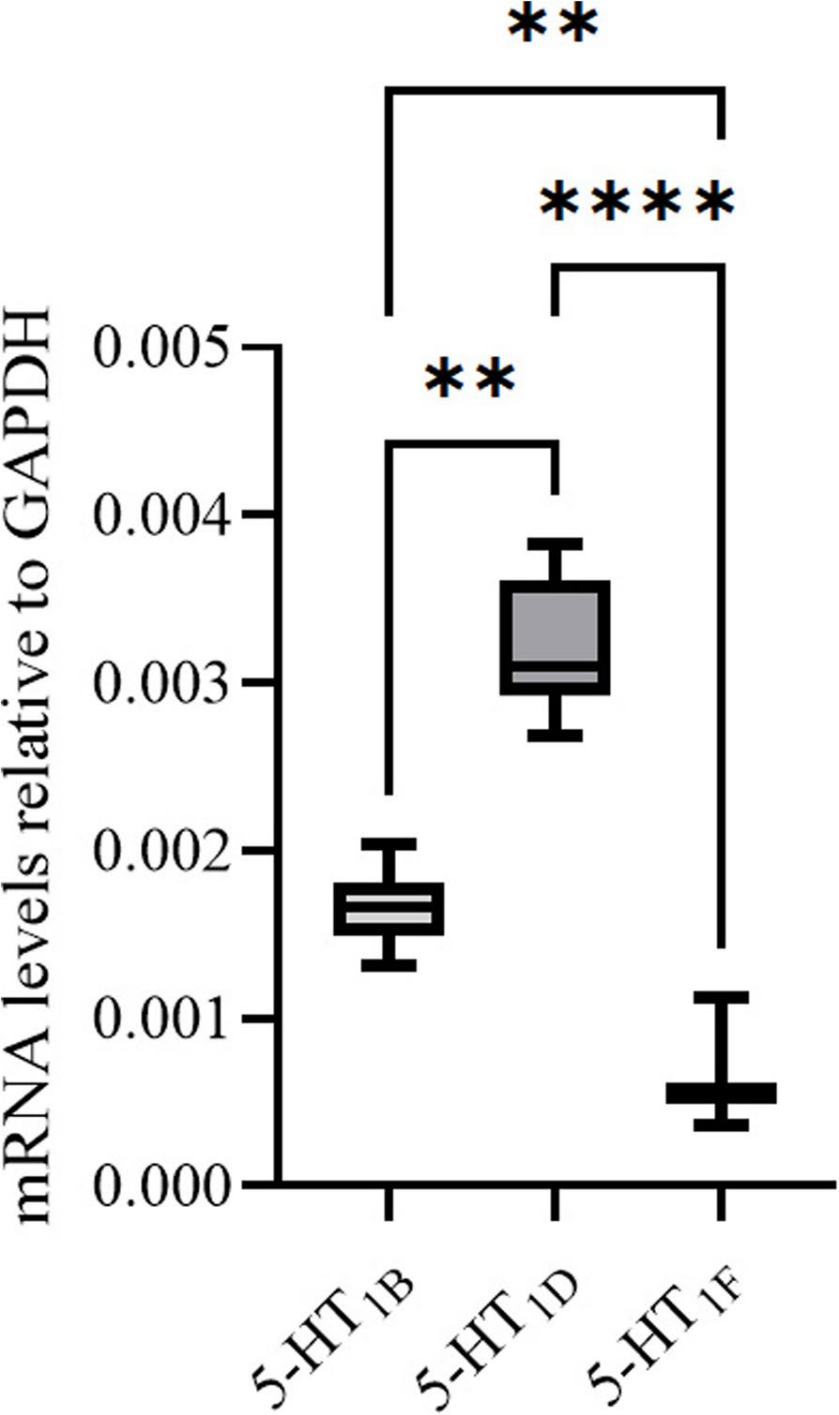
The mRNA expression for 5-HT_1B/1D/1F_ receptors was measured using qPCR in rat TG. The experiments confirmed the expression of all three 5-HT_1_ receptor genes. The 5-HT_1D_ receptor was the most prominently expressed, followed by 5-HT_1B_ and lastly the 5-HT_1F_ receptor. The mRNA levels are shown as relative to the housekeeping gene GAPDH. Data is expressed as mean ± SEM and n = 8. The data was further analysed with Kruskal Wallis non-parametric test with Dunn`s post-hoc test. *P<0.05, **P<0.01 and ***P<0.001

The mRNA expression of 5-HT_1_ receptor subtypes were normalized to the housekeeping gene GAPDH (Fig. 7). The experiments confirmed the mRNA expression of the 5-HT_1B_ receptor (Ct: 23.52 ± 0.16), the 5-HT_1D_ receptor (Ct: 22.57 ± 0.15) and the 5-HT_1F_ receptor (Ct: 25.08 ± 0.41). Statistical analysis revealed significant differences between all three receptor subtypes. The 5-HT_1D_ receptor was the most prominently expressed, followed by the 5-HT_1B_ receptor and lastly the 5-HT_1F_ receptor. In each qPCR experiment either a no template control or a minus reverse transcriptase control was included, and no signs of contamination or genomic DNA were detected in those samples.

### Clinical dose of Lasmiditan in relation to pEC_50_

**Fig. 8:**
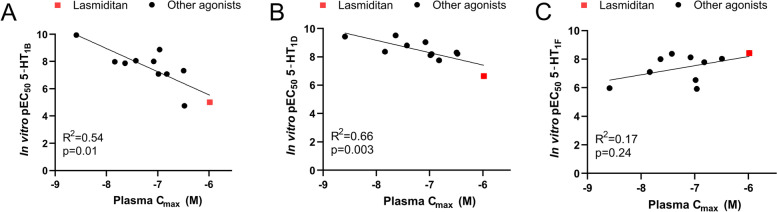
Correlation data for in vitro pEC_50_ versus the clinical plasma concentration C_max_ for multiple triptans and ditans. A significant correlation was observed between the C_max_ and pEC_50_ for the 5-HT_1B_ receptor (**A**), and 5-HT_1D_ receptor (**B**) but not 5-HT_1F_ receptor (**C**). Worth noticing is the correlation being negative for 5-HT_1B_ receptor and 5-HT_1D_ receptor, meaning that the more potent against needed lower C_max_ for clinical effect, opposite to the 5-HT_1F_ receptor correlations. Data is displayed as individual data points with p-values and R^2^ obtained from the correlation analysis. The pEC_50_ data points are from [23] and detailed references and data points for the C_max_ can be found in Supplementary Table 1

Based on the relatively low expression of the 5-HT_1F_ receptor in our experiments we questioned whether the full effect of Lasmiditan occurs via the 5-HT_1F_ receptor or if the recommended dose would allow for a less specific binding to 5-HT_1B/D_ receptors instead. We used publicly available data on plasma concentrations of the current available 5-HT_1_ receptor agonists and correlated this data to the pEC_50_ from a cyclic adenosine monophosphate (cAMP) assay [23]. For both the 5-HT_1B_ (Fig. 8A, R^2^ = 0.54, p = 0.01, n = 11) and the 5-HT_1D_ (Fig. 8B, R^2^ = 0.66, *n* = 11, *p* = 0.003) receptors the data correlated well and as expected for a targeted treatment, higher pEC_50_ nicely matched with a lower plasma concentration. In contrast to 5-HT_1B_ and 5-HT_1D_, the pEC_50_ for the 5-HT_1F_ receptor did not correlate significantly with the plasma concentration (R^2^ = 0.17, *n* = 10, *p* = 0.24), and if anything the correlation was negative (Fig. 8C). Interestingly, Lasmiditan (the red square) is located on the linear trend of both 5-HT_1B_ and 5-HT_1D_ receptors suggesting that the plasma concentration achieved in the clinical studies is high enough to activate these receptors. These results suggest that a considerable part of the anti-migraine effect of Lasmiditan is mediated through 5-HT_1B_ and/or 5-HT_1D_ receptor activation.

### CGRP release experiments and the effect of Lasmiditan

**Fig. 9:**
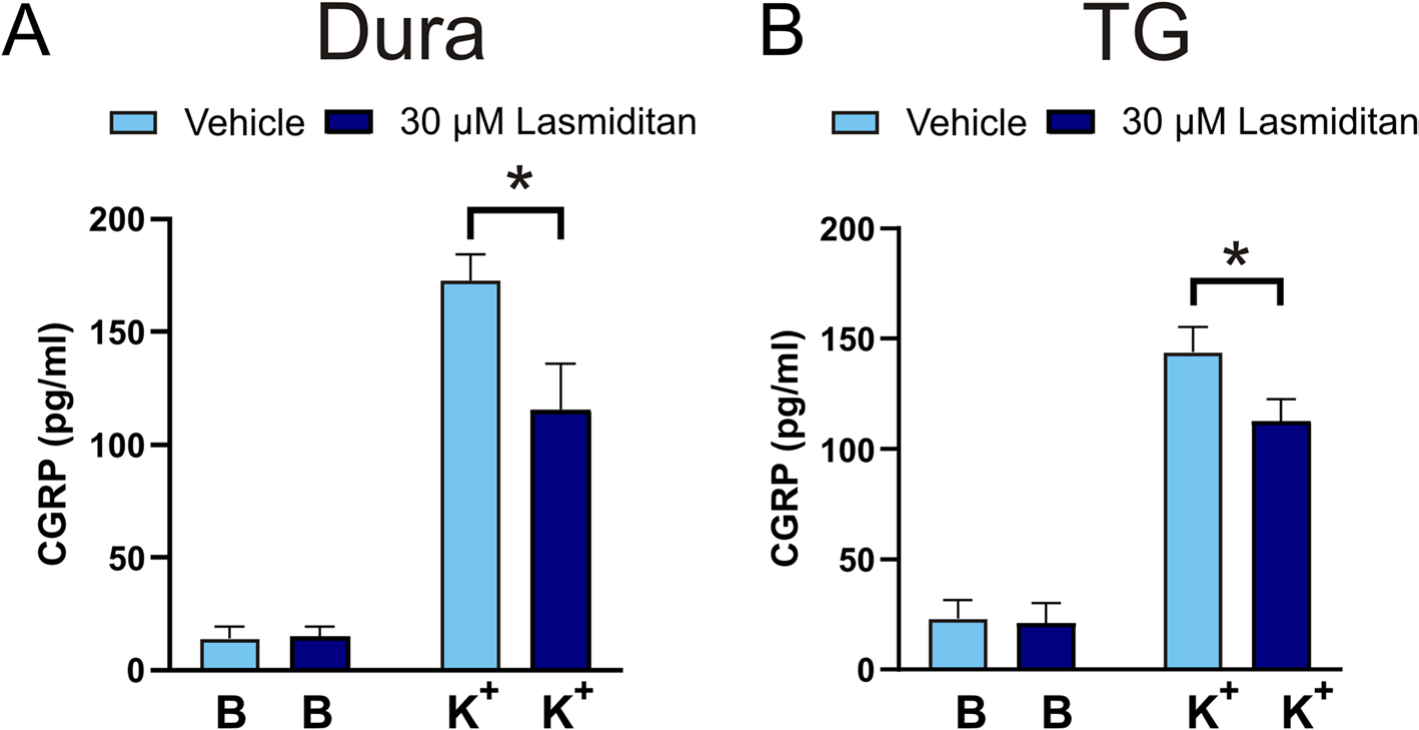
Effect of Lasmiditan on stimulated CGRP release. The addition of 60 mM KCl (K^+^) caused CGRP release from (**A**) the dura (*n* = 5) and (**B**) from TG (*n* = 5). The CGRP release could be inhibited by 30 μM Lasmiditan in both the dura and TG. Data are shown as mean ± SEM with * *p*>0.05, from the paired Student’s T-test being depicted in the graph

To confirm that Lasmiditan also inhibits CGRP release in the hemi-skull model in rats similar to in mice [24], the cranial cups were pre-treated with 30 μM Lasmiditan and stimulated with 60 mM K^+^ (Fig. 9). Similar to in the mice model, Lasmiditan inhibited CGRP release significantly in the dura (Fig. 9A, 172 ± 11 pg/mL vs 115 ± 20 pg/mL, *n* = 5, *p* = 0.032) and in the TG (Fig. 9B, 144 ± 11 pg/mL vs 113 ± 10 pg/mL, *n* = 5, *p* = 0.039).

**Fig. 10:**
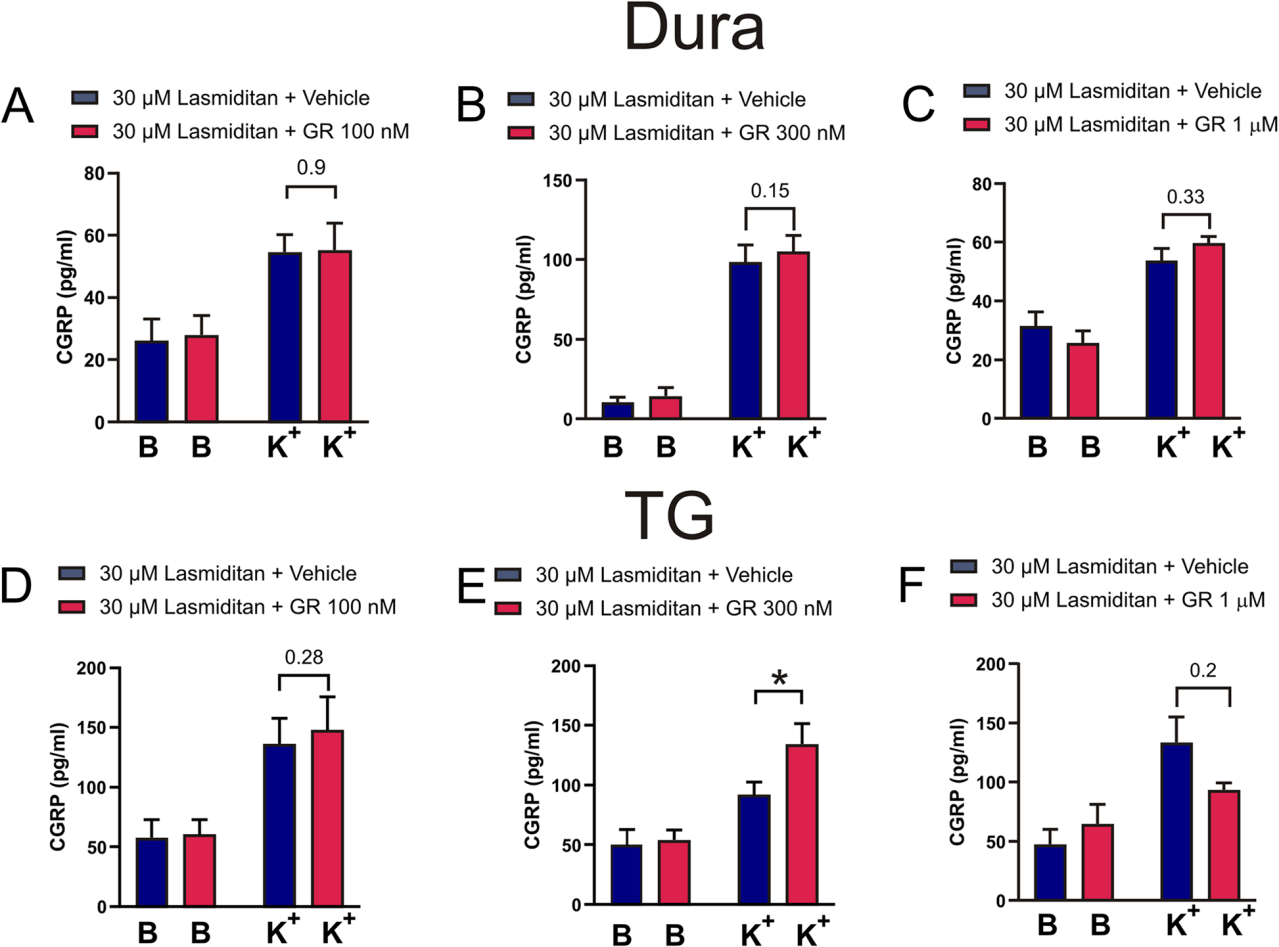
The involvement of 5-HT_1B_ and 5-HT_1D_ receptors on the inhibitory effect of Lasmiditan. Ten minutes before the addition of 30 μM Lasmiditan: (**A**) 100 nM (*n*=4), (**B**) 300 nM (*n*=6) or (**C**) 1 μM (*n*=5) of GR127935, a 5-HT_1B_/5-HT_1D_ blocker was added to the dura which subsequently stimulated with 60 mM KCl (K^+^). Similarly, 10 minutes before the addition of 30 μM Lasmiditan, and before the addition of 60 mM KCl (K^+^) (**D**) 100 nM (*n* = 5), (**E**) 300 nM (*n* = 16) or (**F**) 1 μM (*n* = 6) of GR127935, was added to the TGs which subsequently stimulated with 60 mM KCl (K^+^). The reduction in CGRP release caused by Lasmiditan could be inhibited by 300 nM GR127935 in the TG (*p* = 0.014), with a tendency observed in the dura at the same concentration. Data are shown as mean ± SEM with * p>0.05, from the paired Student’s T-test being depicted in the graph

GR127935, a mixed 5-HT_1B/1D_ antagonist, was added at different concentrations to evaluate if blocking these receptors would counter-act the inhibitory effect of Lasmiditan on CGRP release. For the data on the dura, we did not see any effect at 100 nM GR127935 (Fig. 10A), however we observed a weak effect at 300 nM GR127935 (Fig. 10B, 98 ± 10 pg/mL vs 105 ± 10 pg/mL, *n* = 6, *p* = 0.15), but increasing the concentration did not augment this effect (Fig. 10C).

In the TG, 100 nM of GR127935 did not show significant effect (Fig. 10D, 132 ± 22 pg/mL vs 148 ± 28 pg/mL, *n* = 5, *p* = 0.28). 300 nM showed significantly restored CGRP release (Fig. 10E, 92 ± 11 pg/mL vs 134 ± 17 pg/mL, *n* = 16, *p* = 0.014), whereas 1 μM of GR 127935 had no effect (Fig. 10F, 134 ± 21 pg/mL vs 93 ± 6 pg/mL, *n* = 6, *p* = 0.2). If anything, GR127935 had an additional inhibitory effect suggesting it works as a partial agonist at the high concentration, as has been documented [34], or potentially interfering with the 5-HT_3_ receptor system [35]. GR127935 was further evaluated on its own and no effects *per se* were observed (Suppl. Fig. 1).

As we observed the strongest correlation of our data on the 5-HT_1D_ receptor, BRL15572, a 5-HT_1D_ specific antagonist, was also tested at 1.25 μM (Suppl. Fig. 2). This antagonist produced an additional inhibition of CGRP release in the dura (Suppl. Fig. 2A, 91 ± 17 pg/mL vs 64 ± 6 pg/mL, *n* = 5, *p* = 0.046). BRL15572 is likely acting as a partial agonist [34], but showed no effect in the TG (Suppl. Fig. 2B, 145 ± 11 pg/mL vs 126 ± 13 pg/mL, *n* = 5, *p* = 0.34), however this was not pursued further.

### Release of 5-HT from the TVS

**Fig. 11:**
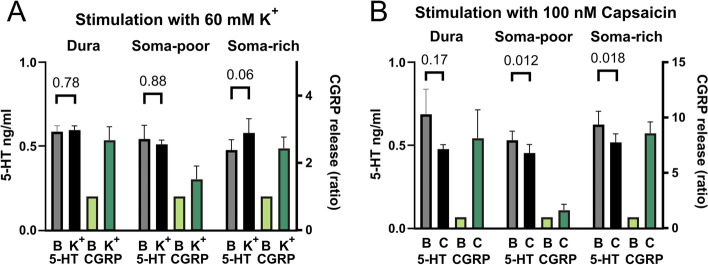
Stimulated release of 5-HT and CGRP in TG and dura mater. **A** The addition of 60 mM KCl (K^+^) caused no 5-HT release from the dura (*n* = 6) or soma-poor TG (*n* = 5), but a significant release was seen from the soma-rich TG (*n* = 6). **B** The addition of 100 nM capsaicin caused no 5-HT release from the dura (*n* = 7), soma-poor (*n* = 5) or soma right TG (*n* = 6). In contrast a significant reduction in 5-HT was observed for both the soma-poor and soma-rich TG. For all the samples CGRP content was also measured and used for the correlation analysis. Data are shown as mean ± SEM or their individual data points with pairing, and with *p* values obtained with Student’s T-test being depicted in the graph

Although the most commonly used anti-migraine drugs are targeting the 5-HT signaling system, nearly no data on its intrinsic role in the TG exist. The IHC data revealed the presence of several 5-HT-ir fibres in the TG. The study therefore opted to investigate potential 5-HT release from the TVS (Fig. 11). Release was stimulated with 60 mM K^+^ (Fig. 11A) or 100 nM Capsaicin (Fig. 11B). The CGRP release data was collected in parallel as a comparative. 5-HT was observed above the detection limit in all samples, but was not found to be released from the dura (0.59 ± 0.04 ng/mL to 0.60 ± 0.03 ng/mL, *n* = 6, *p* = 0.78) nor was it released from the soma-poor TG (0.54 ± 0.08 ng/mL to 0.51 ± 0.03 ng/mL, *n* = 5, *p* = 0.88). 5-HT was only detected to be released at levels higher than baseline in the soma-rich TG (0.48 ± 0.06 ng/mL to 0.58 ± 0.08 ng/mL, *n* = 6, *p* = 0.06), which coincidentally is where most of the 5-HT-ir C-fibres were observed. For the data on stimulation with capsaicin, the 5-HT levels were slightly lower in the dura (0.68 ± 0.15 ng/mL to 0.48 ± 0.03 ng/mL, *n* = 7, *p* = 0.17), soma-poor TG (0.53 ± 0.05 ng/mL to 0.45 ± 0.05 ng/mL, *n* = 5, *p* = 0.012) and soma-rich TG (0.62 ± 0.08 ng/mL to 0.52 ± 0.05 ng/mL, *n* = 6, *p* = 0.018). In the soma-rich TG there was a significantly higher 5-HT release following the 60 mM K^+^ compared to the 100 nM Capsaicin (0.58 ± 0.08 ng/mL vs 0.52 ± 0.05 ng/mL, *n* = 6, *p* = 0.02).

**Fig. 12:**
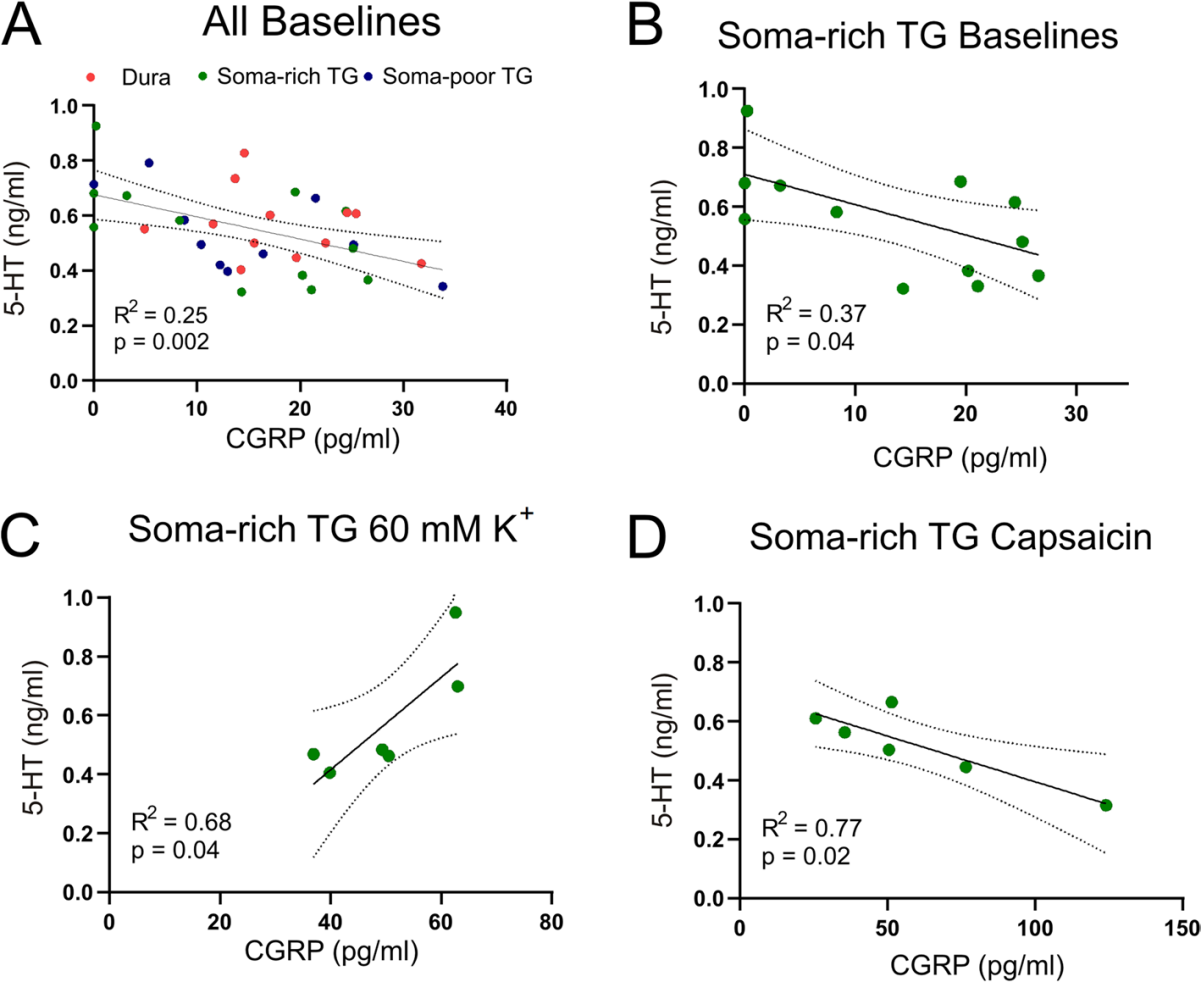
Correlation of *in situ* 5-HT and CGRP release from the same dural or trigeminal ganglion samples. **A** Correlations of constitutive release (all baseline measurements) from the dura, soma-poor and soma rich TG. A significant negative correlation was observed. **B** Correlations of constitutive release from the soma-rich TG only, and here a significant negative correlation was observed. **C** The stimulated release (addition of 60 mM KCl (K^**+**^) or (**D**) 100 nM capsaicin, of 5-HT and CGRP from the same soma-rich TGs. Significant positive correlation was observed for K^+^ which contrasts to negative correlation observed with capsaicin. Data are shown as individual data points with p values and R^2^ obtained from the correlation analysis

The 5-HT concentrations were correlated with the CGRP concentrations in the parallel samples. Interestingly, the 5-HT concentration showed significantly negative correlation (Fig. 12A, R^2^ = 0.25, *n* = 34, *p* = 0.002) with CGRP in all baseline samples. This indicates that 5-HT is a natural inhibitor of basal CGRP levels. Although the correlation holds for all samples being combined, the trend was not significant for the dura (R^2^ = 0.07, *n* = 12, *p* = 0.42), and only near significant for the soma-poor TG (R^2^ = 0.37, *n* = 10, *p* = 0.06) by themselves. In contrast, the correlation for the soma-rich TG was found to be significant (Fig. 12B, R^2^ = 0.37, *n* = 12, *p* = 0.04). For the 60 mM K^+^ stimulation and 100 nM Capsaicin, we did not observe any significant correlation when all samples were combined. However, significant positive correlation with 60 mM K^+^ simulation (Fig. 12C, R^2^ = 0.68, *n* = 6, *p* = 0.04) was observed for the soma-rich TG, where 5-HT released was detected, suggesting the possibility of a negative feedback on the CGRP release. This contrasts to the data on capsaicin, here the data negatively correlate (Fig. 12D, R^2^ = 0.77, *n* = 6, *p* = 0.02), suggesting that capsaicin stimulation potentially stimulates 5-HT uptake or breakdown.

## Discussion

This study reveals the expression of 5-HT-ir throughout in the rat TG and most interestingly in a subpopulation of C-fibres lacking both CGRP- and SP-ir. In addition, we have demonstrated the differential expression of the 5-HT_1B/1D/1F_ receptors in the TVS, which markedly add to understanding of the clinical responses to triptans and ditans.

The immunohistochemical findings reveal that 5-HT in the TG seems to be robustly expressed in a small subset of thin C-fibres and in the majority of neuron cell bodies. A weak and often patchy ir for 5-HT was also found in Aδ-fibres, Schwann cells and SGCs. Interestingly, the C-fibres expressing 5-HT were more frequently found near Redlich-Obersteiner’s zone and was rarely found in distal parts of the TG, suggesting that the fibres may be projecting from the brainstem and terminating in the TG. The 5-HT-ir C-fibres presented varicosities and were often markedly thinner than the observed CGRP and SP-ir in C-fibres. This has, to our knowledge, only been observed previously in cat TG, where the thin fibres were remarkably found to encircle some TG neurons [36].

The varied staining intensity and sporadic expression of 5-HT in the Aδ-fibres and non-neuron cells support a potential hypothesis of 5-HT reuptake released from e.g. plasma, platelets and mast cells [37]. This agrees well with previous studies reporting low levels of TPH [29, 30]. Although we found TG neurons and fibres expressing 5-HT, it is to the best of our knowledge not known if these cells can release 5-HT in the TG. To gain further insight into this question, we performed stimulated release experiments on 5-HT and CGRP (Fig. 11). We were only able to trigger 5-HT release from the soma-rich part of the TG, which interestingly is where most 5-HT containing C-fibres were observed. Furthermore, it is plausible that the relatively high 5-HT baseline may mask the presumed stimulated release from the subset of thin C-fibres ir to 5-HT, which likely is quite low given the small number and diameter of these fibres.

The 5-HT_1D_ receptor expression was observed in neuron cell bodies, often presented with a clear nuclear staining, and in Aδ-fibres, where it co-localized with RAMP1. No 5-HT_1D_ receptor ir was detected in CGRP-positive C-fibres. In contrast, the 5-HT_1F_ receptor ir was only faintly observed in some neuron cell bodies. This low expression was confirmed with semi quantitative light emission measurements where 5-HT_1F_ ir did not significantly differentiate from the negative control (Fig. 1) and showed a sparse expression in the qPCR analysis (Fig. 7).

5-HT_1B_ receptor expression was found in neuron cell bodies and to some extent in Aδ-fibres, in accordance with previous studies utilizing the same antibodies [38]. The Aδ-fibre expression of the 5-HT_1B_ receptor was confirmed by the co-localization of RAMP1 and CASPR marking the same fibre [39]. The weak 5-HT_1B_ receptor expression in Aδ-fibres may indicate that the more dominantly expressed 5-HT_1D_ receptor may be responsive for the triptan counteraction on the CGRP activated cAMP-pathway in TG Aδ-fibres [40, 41]. However, the high expression of 5-HT_1B_ and 5-HT_1D_ receptors in whole TG samples observed in qPCR analysis agrees with previous work and is presently elaborated with their robust expression in neuron somas and Aδ-fibres.

The 5-HT_1_ receptor mRNA expression analysis revealed that 5-HT_1D_ receptor mRNA was more prominently expressed than 5-HT_1B_ and 5-HT_1F_, which is comparable to results from previous studies in rats [42] and data from humans, where the 5-HT_1F_ receptor expression is 90% less than 5-HT_1B_ [43]. Some of the aspects of the current study were addressed using LY-344864 (an earlier 5-HT_1F_ receptor agonist) by Amrutkar and colleagues [42], but there are some important differences. Firstly, the study did not find significant CGRP release inhibition in the TG by LY-344864, and secondly the effect of a 5HT_1B_/5HT_1D_ receptor antagonist was therefore not evaluated. Similar to our study LY-344864 had inhibitory effect in the dura, and they could not show significant restoration of CGRP release by GR127395. This opens up the possibility that there is a difference in the dural release, plausibly via terminal synapses, when compared the release from the TG.

The light emission data (Fig. 1A) for 5-HT_1F_ receptors did not differ from the negative control. Taken together with the sparse immunohistochemical expression and the relatively low mRNA level, suggests that the concentration of the 5-HT_1F_ receptor is considerably lower than 5-HT_1B_ and 5-HT_1D_ receptors in the rat TG and agrees well with the low expression of 5-HT_1F_ receptors in the Aδ-fibres.

While we found co-localization of 5-HT and CGRP in neuron somas, we did not detect any co-localization of 5-HT and CGRP in C-fibres. Similarly, we did not find 5-HT-ir in the axon hillocks of CGRP positive neurons. This may indicate that the observed thin unmyelinated 5-HT positive C-fibres have a different origin as compared to the more classical C-fibres that store CGRP and SP. In the early literature this has been discussed and lesion to the raphe neurons revealed that perivascular microvascular 5-HT fibres may in part originate from these central neurons [44, 45], however this question should in relation to the role of 5-HT in the trigeminal system be addressed in future studies.

The most exciting implication of this study would suggest that 5-HT has a physiological signalling role in the TVS and could be released from neuron cell bodies and fibres. In addition to the fibres of 5-HT that we observed, there could also be other sources of 5-HT. Chan-Palay reported that experimentally labelled 5-HT can be taken up by varicose fibres innervating cerebral blood vessels [46]. Similarly, medullary neurons proximal to large medullary arteries have also been found to contain 5-HT and hypothesized to be chemosensitive [47]. Another aspect is that brain micro vessels were found to contain perivascular 5-HT originating in the raphe nuclei in the brainstem [45]. Importantly our work provides the answer to the long-lasting question, of why are there 5-HT_1_ receptors in the TVS? Showing that 5-HT negatively correlate with the baseline CGRP levels, now we know, that it is most likely not like people decades ago said in relation to discovering triptans, “they are there for us to treat migraine”, but that there is a serotonergic signalling system within the TVS, and that 5-HT receptors have a physiological role.

The results from the 12-month long GLADIATOR study displayed evidence for reduction in headache days and improved MIDAS scores for participants [48]. However, the authors noted a substantial discontinuation of Lasmiditan treatment throughout the study. 42.1% of the discontinuations were due to “patient request” [49] which comprised e.g. lack of efficacy, dislike of the investigational product or initiation of prohibited medication, among others. It should however be noted that the GLADIATOR study design restricted the patients from driving up to 12 h after dosing, which likely reduced compliance further. FDA has this now as a note in prescription details for use of Lasmiditan.

Further, a study found almost no correlation between increasing lipophilicity of triptans and their therapeutic gain, while the authors noted an almost significant correlation between increasing lipophilicity and CNS adverse events [50]. This suggests that CNS penetrating anti-migraine drugs will have the disadvantage of CNS adverse events (e.g. dizziness). Neither triptans, gepants or monoclonal antibodies (mAbs) can easily cross the BBB or its functional equivalent the blood-spinal cord barrier [51, 52]. However, there are animal studies indicating that triptans may inhibit secondary neurons in the trigeminal nucleus caudalis (TNC) [21, 53, 54]. It remains debated if this occurs in vivo at therapeutic concentrations.

In contrast, the recently introduced drug-class ditans have been shown to cross the BBB in the recommended dose and could thus exert its effect centrally in addition to the periphery. Nevertheless, it is most likely that the inhibitory effect of Lasmiditan on CGRP release occurs in the periphery, and that its lesser effect is to selectively activate the 5-HT_1F_ receptors at secondary neurons inhabiting the TNC within the upper spinal cord. Interestingly, when correlating the plasma concentration needed for effect of Lasmiditan, it aligns with the triptans for the 5-HT_1B_ and 5-HT_1D_ receptors (Fig. 8). This in our opinion, combined with the effect of GR127934 (Fig. 10) suggest that part of the anti-migraine effect of Lasmiditan is inhibition of CGRP release through “unspecific” activation of 5-HT_1B_ and 5-HT_1D_ receptors in the TG.

As shown in this study, co-localization of CGRP with 5-HT_1B/1D_ receptors was not observed in trigeminal C-fibres, but rather in neuron somas. This data implies that the inhibitory action of triptans on CGRP release is not the same in the dura mater and the TG. This has been previously suggested [55, 56] and implicates the TG as a treatable target in the processing of migraine pain. Still, there is data showing that sumatriptan can inhibit CGRP release in both dura mater and “soma-rich” TG [39]. Therefore, it could be that the C-fibres just express a modest amount of receptors, which we cannot detect microscopically. Nevertheless, it raises the question of whether the triptans work the way we currently believe or if neuronal/glial cross talk plays a larger part than previously expected.

**Fig. 13:**
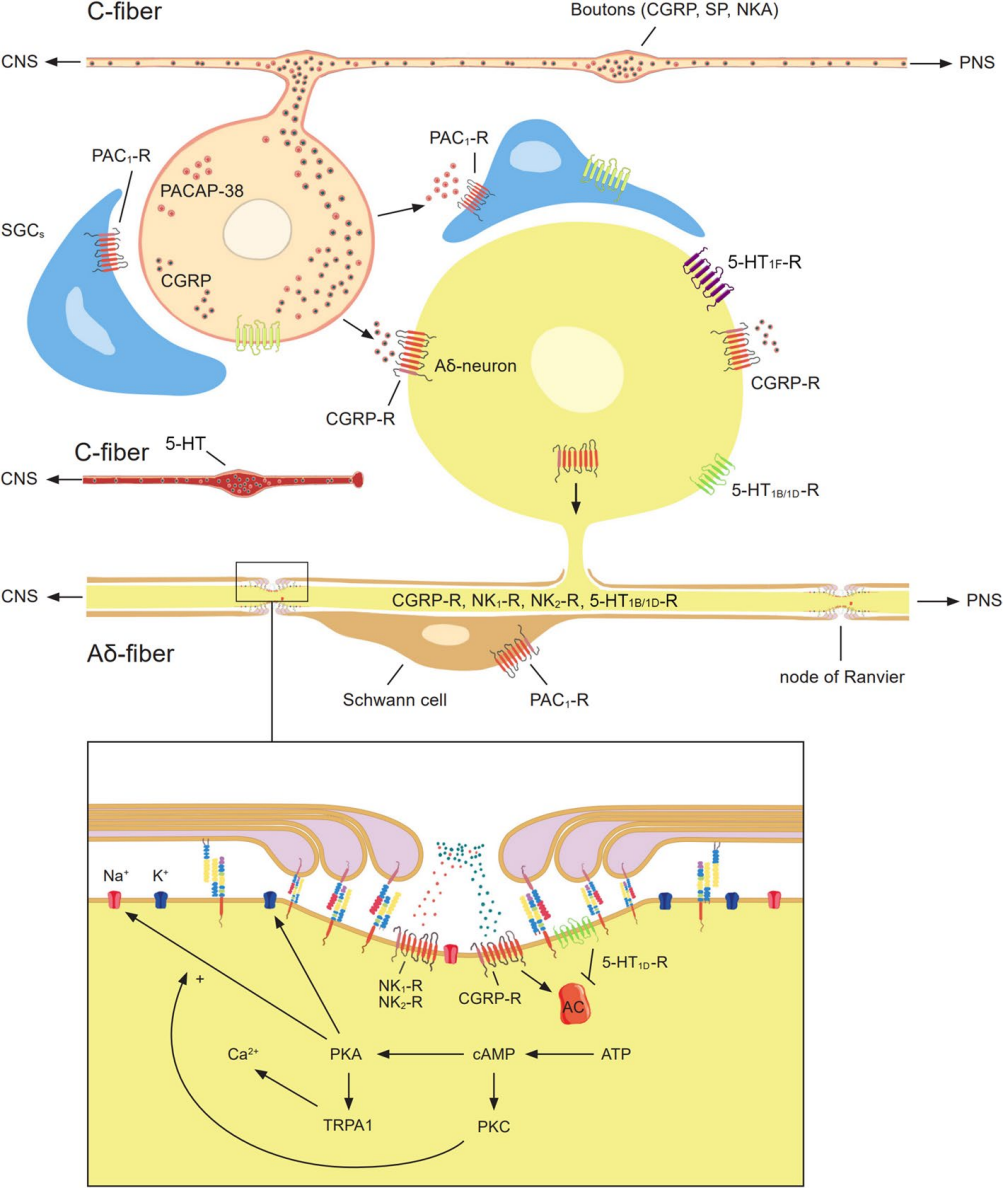
Simplified overview of immunoreactivity within the rat trigeminal ganglion. The schematic is based on observations made in this, and previous studies [11, 39, 62]. **A** Expression of 5-HT, CGRP, neurokinin and PACAP-38 signalling elements in trigeminal neurons and glial cells. **B** Hypothetic CGRP and neurokinin receptor signalling within the nodes of Ranvier of trigeminal Aδ-fibres. Abbreviations; neurokinin A (NKA), neurokinin receptor 1 (NK_1_-R), neurokinin receptor 2 (NK_2_-R), peripheral nervous system (PNS), transient receptor potential ankyrin subtype 1 (TRPA1)

Crosstalk between trigeminal neurons and SGCs has been proposed to occur via gap junctions, cytokines, purinergic signalling, glutamate signalling, and others reviewed elsewhere [57]. Purinergic signalling was first proposed to play a role in migraine pain by Burnstock [58, 59], and it has been suggested that increased extracellular ATP activates trigeminal nociceptors via P2X3 receptors [60]. Purinergic signalling from neuron to glial cells could be disrupted by activation of the cAMP dependent pathway via CGRP or neurokinin signalling in the trigeminal system. This could occur through intracellular reduction/depletion of ATP mediated by the adenylyl cyclase driven formation of cAMP. Simultaneously, CGRP receptor activation drives the biosynthesis of ATP, ADP and AMP, while reducing internal stores of adenosine, which has been observed in cultured trigeminal neurons and meninges [61]. The same would be true for pituitary adenylate cyclase-activating polypeptide 38 (PACAP-38), although we found no clear pituitary adenylate cyclase-activating polypeptide receptor (PAC_1_-R) expression in neurons [62]. Instead the PAC_1_-R was observed mainly in SGCs surrounding neurons, implying a reversed relationship in this signaling system. Furthermore, it is possible that the activation of observed 5-HT_1B_ receptors in SGCs modulates adenylyl cyclase activated by the PAC_1_-R. As 5-HT_1B_ receptor activation is inherently inhibitory, it could, in addition, exert an effect on other pain-related factors (e.g. cytokines) in the crosstalk between neuron and glia. Investigations of novel intercellular signalling mechanism or disruption of an ongoing feedback loop should be addressed in future studies. A simplified overview of our recent observations is presented in Fig. 13.

The current study has some limitations; Firstly, we cannot be sure that the expression data will match that of human subjects as this can vary between species meaning that our study may not necessarily reflect that of a human specimen. However, an earlier study on the expression of 5-HT_1B/1D_ receptors in human trigeminal ganglion shows, in agreement with our present study, that 5-HT_1B/1D_ receptors are co-localized with CGRP and SP in neuron somas but no expression in axons was reported [20]. The receptor expression matches well with the clinical data observed, such as the plasma concentration needed to for clinical effect (Fig. 8). The major limitation is the study being performed in young and healthy animals, and could differ from migraineurs, but could highlight important biological mechanisms. One such mechanisms is the question being raised in the current paper of the potential of direct inhibition of Aδ-fibers by 5-HT_1D_ receptors. Our data supports such an idea, but studies in human subjects would be needed to support the hypothesis. We believe that revisiting more specific 5-HT_1D_ receptor agonists could be a relevant approach.

In conclusion, the 5-HT_1F_ receptor is weakly expressed in the TVS compared with 5-HT_1B_ and 5-HT_1D_ receptors. Further, data from our rodent studies combined with analysis of pEC_50_ and C_max_ from the literature, show that parts of the anti-migraine effect of Lasmiditan could occur through activation 5-HT_1B/D_ receptors. As the 5-HT_1D_ receptor does not strong vasocontractile effects, we suggest that a specific 5-HT_1D_ receptor active drug should be revisited. Lastly, we hypothesize that 5-HT_1B/1D_ receptor activation could lead to both the inhibition of CGRP release and the inhibition of the cAMP dependent pathway in trigeminal Aδ-fibres.

## Supplementary Information

**Additional file 1: Supplementary Figure 1.** Involvement of the 5-HT_1B_ and 5-HT_1D_ receptors in the effect of Lasmiditan. Before the addition of 30 μM Lasmiditan, 300 nM of GR127935, a 5HT_1B_/5HT_1D_ blocker was added to the TG which subsequently stimulated with 60 mM KCl (K^+^). Data are shown as mean ± SEM with * *p*>0.05, from the paired Student’s T-test being depicted in the graph.

**Additional file 2: Supplementary Figure 2.** Involvement of the 5-HT_1D_ receptors in the effect of Lasmiditan. Before the addition of 30 μM Lasmiditan, 1.25 μM of BRL15572, a 5HT_1D_ receptor antagonist was added to the dura (*n* = 5) which subsequently stimulated with 60 mM KCl (K^+^). Similarly, before the addition of 60 mM KCl (K^+^) 1.25 μM (*n*=5) of BRL15572, was added to the TGs which subsequently stimulated with 60 mM KCl (K^+^). BRL15572 significantly inhibited CGRP release further in the dura (*P*=0.0456) with a tendency observed in the TG at the same concentration. Data are shown as mean ± SEM with * *p*>0.05, from the paired Student’s T-test being depicted in the graph.

**Additional file 3: Supplementary Table 1.** Data points used for correlation in Fig. 8.

## Data Availability

The datasets generated during and/or analysed during the current study are available from the corresponding author on reasonable request.

## Abbreviations

*5-HT*: 5-Hydroxytryptamine
*5-HT*_*1B*_: 5-Hydroxytryptamine receptor 1B
*5-HT*_*1D*_: 5-Hydroxytryptamine receptor 1D
*5-HT*_*1F*_: 5-Hydroxytryptamine receptor 1F
*BBB*: Blood-brain barrier
*BSA*: Bovine serum albumin
*cAMP*: Cyclic adenosine monophosphate
*CASPR*: Contactin associated protein 1
*CGRP*: Calcitonin gene-related peptide
*CNS*: Central nervous system
*C*_*t*_: Cycle threshold
*DMSO*: Dimethyl sulfoxide
*ELISA*: Enzyme-linked immunosorbent assay
*GAPDH*: Glyceraldehyde-3-phosphate dehydrogenase
*IHC*: Immunohistochemistry
*Ir*: Immunoreactivity
*MAbs*: Monoclonal antibodies
*MBP*: Myelin basic protein
*NKA*: Neurokinin A
*NK*_*1*_*-R*: Neurokinin receptor 1
*NK*_*2*_*-R*: Neurokinin receptor 2
*PACAP-38*: Pituitary adenylate cyclase-activating polypeptide 38
*PAC*_*1*_*-R*: Pituitary adenylate cyclase-activating polypeptide receptor
*PBS*: Phosphate buffered saline
*PBS-T*: Phosphate buffered saline with Triton X-100
*PNS*: Peripheral nervous system
*qPCR*: Quantitative polymerase chain reaction
*RAMP1*: Receptor activity modifying protein 1
*SGCs*: Satellite glial cells
*SP*: Substance P
*SIF*: Synthetic interstitial fluid
*TG*: Trigeminal ganglion
*TNC*: Trigeminal nucleus caudalis
*TPH*: Tryptophan hydroxylase
*TRPA1*: Transient receptor potential ankyrin subtype 1
*TVS*: Trigeminovascular system
