# Supplementary figures and images for: Lasmiditan and 5-Hydroxytryptamine in the rat trigeminal system; expression, release and interactions with 5-HT1 receptors
Source: J Headache Pain. 2022 Feb 17;23(1):26. doi: 10.1186/s10194-022-01394-z (PMC8903724; doi:10.1186/s10194-022-01394-z)

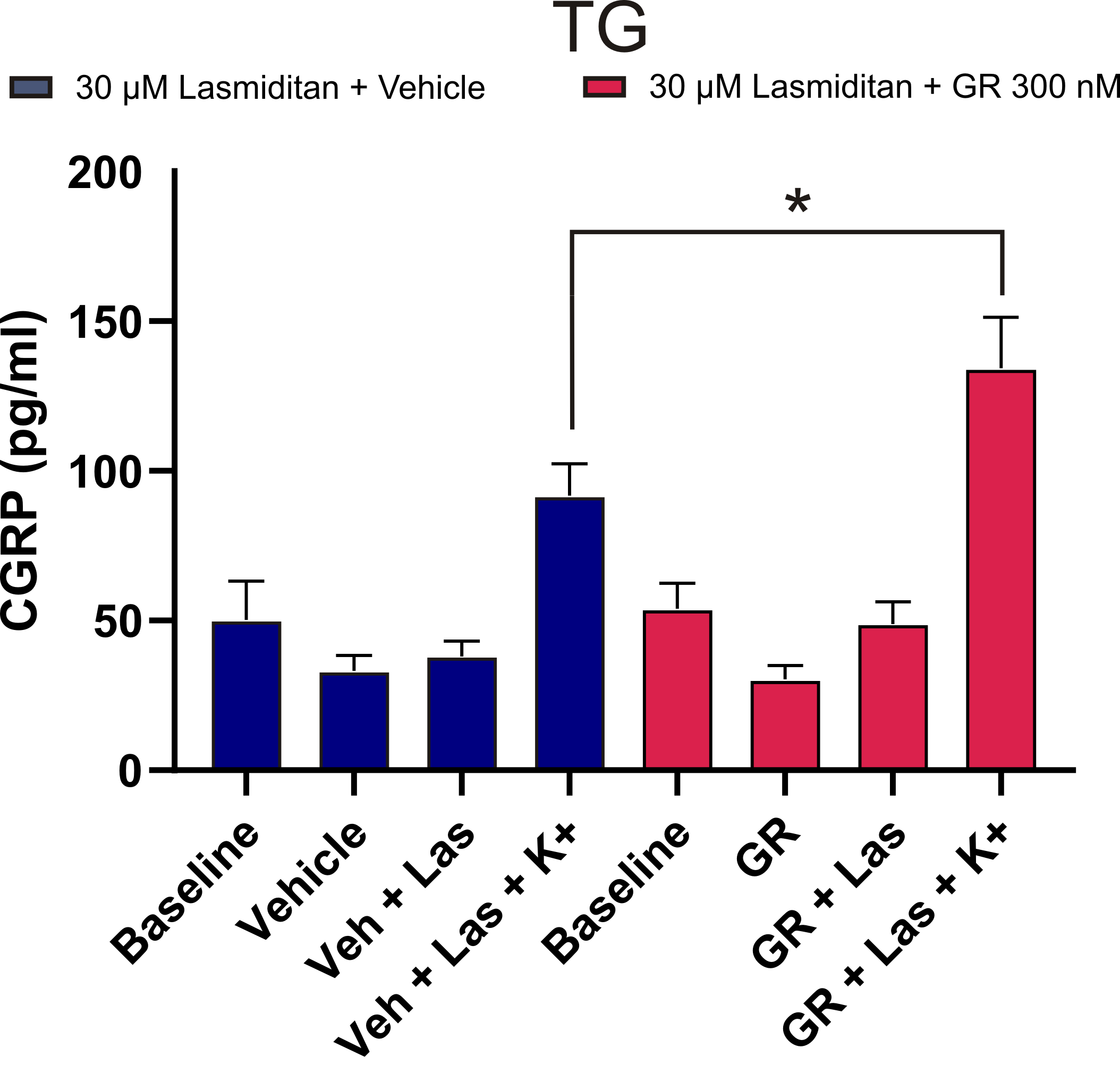

Supplement: Supplementary file 1 — Additional file 1: Supplementary Figure 1. Involvement of the 5-HT1B and 5-HT1D receptors in the effect of Lasmiditan. Before the addition of 30 μM Lasmiditan, 300 nM of GR127935, a 5HT1B/5HT1D blocker was added to the TG which subsequently stimulated with 60 mM KCl (K+). Data are shown as mean ± SEM with * p>0.05, from the paired Student’s T-test being depicted in the graph. [file 10194_2022_1394_MOESM1_ESM.tif]

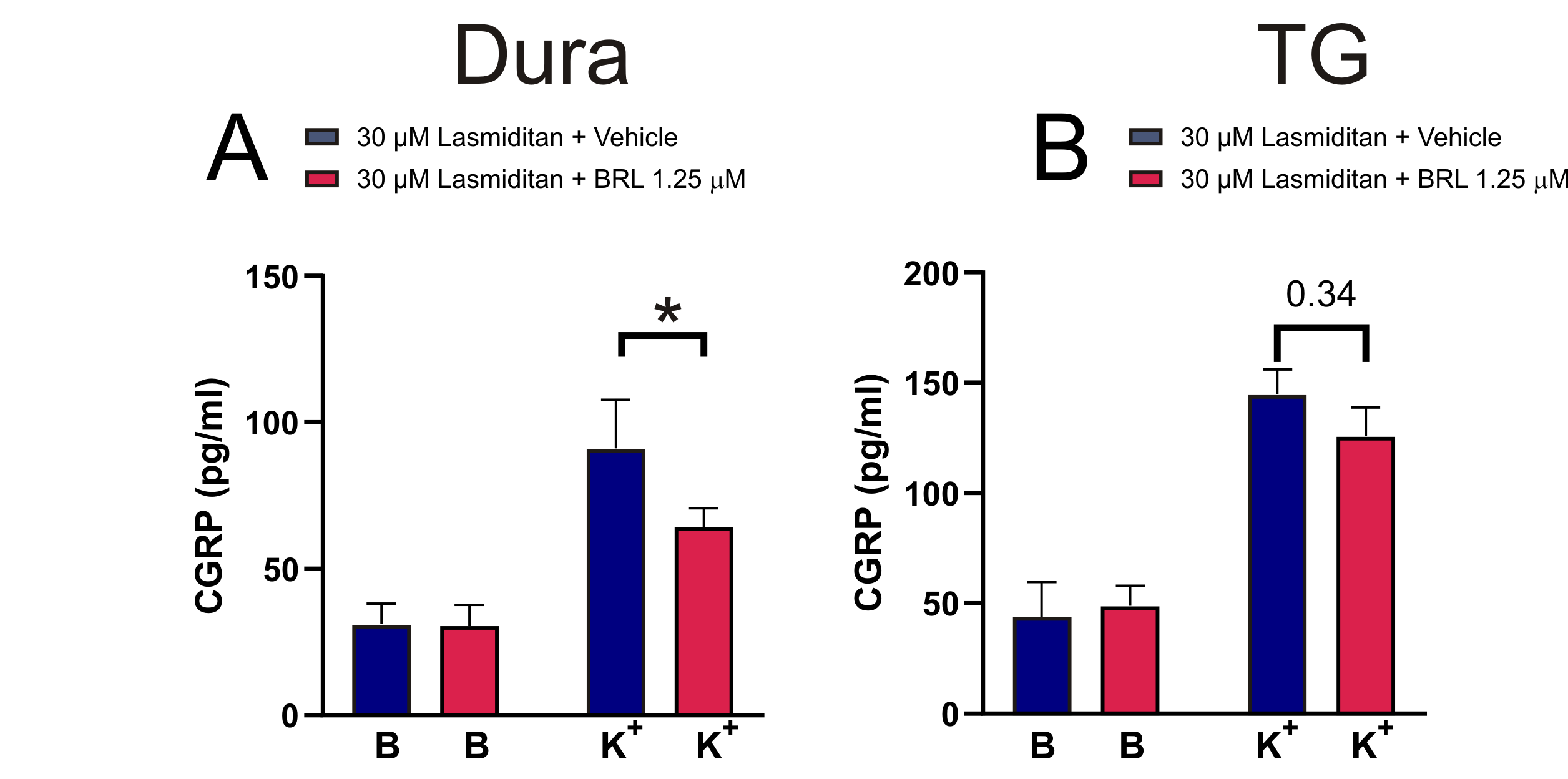

Supplement: Supplementary file 2 — Additional file 2: Supplementary Figure 2. Involvement of the 5-HT1D receptors in the effect of Lasmiditan. Before the addition of 30 μM Lasmiditan, 1.25 μM of BRL15572, a 5HT1D receptor antagonist was added to the dura (n = 5) which subsequently stimulated with 60 mM KCl (K+). Similarly, before the addition of 60 mM KCl (K+) 1.25 μM (n=5) of BRL15572, was added to the TGs which subsequently stimulated with 60 mM KCl (K+). BRL15572 significantly inhibited CGRP release further in the dura (P=0.0456) with a tendency observed in the TG at the same concentration. Data are shown as mean ± SEM with * p>0.05, from the paired Student’s T-test being depicted in the graph. [file 10194_2022_1394_MOESM2_ESM.tif]
