# Supplementary material for: Lasmiditan and 5-Hydroxytryptamine in the rat trigeminal system; expression, release and interactions with 5-HT1 receptors
Source: J Headache Pain. 2022 Feb 17;23(1):26. doi: 10.1186/s10194-022-01394-z (PMC8903724; doi:10.1186/s10194-022-01394-z)
Supplement: Supplementary file 3 — Additional file 3: Supplementary Table 1. Data points used for correlation in Fig. 8. [file 10194_2022_1394_MOESM3_ESM.docx]

|  | C_max_ (M) | pEC_50_ 5HT_1B_ | pEC_50_ 5HT_1D_ | pEC_50_ 5HT_1F_ |
| --- | --- | --- | --- | --- |
| Ergotamine tartrate^1^ | 2.58E-09 | 9.94 | 9.43 | 5.97 |
| Sumatriptan succinate^2^ | 3.22E-07 | 7.32 | 8.3 | 8.03 |
| Zolmitriptan^3^ | 2.31E-08 | 7.87 | 9.51 | 8.0 |
| Naratriptan hydrochloride^4^ | 3.76E-08 | 8.05 | 8.8 | 8.38 |
| Rizatriptan benzoate^5^ | 1.04E-07 | 7.08 | 8.11 | 6.54 |
| Almotriptan malate^6^ | 1.51E-07 | 7.08 | 7.75 | 7.79 |
| Eletriptan hydrobromide^7^ | 8.37E-08 | 8.0 | 9.04 | 8.13 |
| Frovatriptan racemate^8^ | 1.46E-08^a^ | 7.98 | 8.36 | 7.1 |
| Alniditan dihydrochloride^9^ | 1.09E-07 | 8.87 | 8.2 | 5.92 |
| PNU-142633^10^ | 3.34E-07 | 4.74 | 8.22 | N.D |
| Lasmiditan hemisuccinate^11^ | 1.04E-06 | 5.0 | 6.64 | 8.43 |

^a^average male/female

All pEC50 data are from Table 2 in *Rubio-Beltran et al.* analyzing secondary messenger activity^12^.

1 Orton, D. A. & Richardson, R. J. Ergotamine absorption and toxicity. *Postgrad Med J* **58**, 6-11, doi:10.1136/pgmj.58.675.6 (1982).

2 Lacey, L. F., Hussey, E. K. & Fowler, P. A. Single dose pharmacokinetics of sumatriptan in healthy volunteers. *Eur J Clin Pharmacol* **47**, 543-548, doi:10.1007/BF00193709 (1995).

3 Dixon, R. & Warrander, A. The clinical pharmacokinetics of zolmitriptan. *Cephalalgia* **17 Suppl 18**, 15-20, doi:10.1177/0333102497017S1803 (1997).

4 Dulli, D. A. Naratriptan: an alternative for migraine. *Ann Pharmacother* **33**, 704-711, doi:10.1345/aph.18300 (1999).

5 Lee, Y. *et al.* Pharmacokinetics and tolerability of oral rizatriptan in healthy male and female volunteers. *Br J Clin Pharmacol* **47**, 373-378, doi:10.1046/j.1365-2125.1999.00917.x (1999).

6 Ravikumar, K., Chandu, B. R., Challa, B. R. & Chandrasekhar, K. B. Method development and validation of almotriptan in human plasma by HPLC tandem mass spectrometry: application to pharmacokinetic study. *Sci Pharm* **80**, 367-378, doi:10.3797/scipharm.1112-01 (2012).

7 Patel, H. *et al.* Differential pharmacokinetic drug-drug interaction potential of eletriptan between oral and subcutaneous routes. *Xenobiotica* **49**, 1202-1208, doi:10.1080/00498254.2018.1540805 (2019).

8 Elkind, A. H., Wade, A. & Ishkanian, G. Pharmacokinetics of frovatriptan in adolescent migraineurs. *J Clin Pharmacol* **44**, 1158-1165, doi:10.1177/0091270004268046 (2004).

9 Roon, K. I., Soons, P. A., Uitendaal, M. P., de Beukelaar, F. & Ferrari, M. D. Pharmacokinetic profile of alniditan nasal spray during and outside migraine attacks. *Br J Clin Pharmacol* **47**, 285-290, doi:10.1046/j.1365-2125.1999.00894.x (1999).

10 Gomez-Mancilla, B. *et al.* Safety and efficacy of PNU-142633, a selective 5-HT1D agonist, in patients with acute migraine. *Cephalalgia* **21**, 727-732, doi:10.1046/j.1468-2982.2001.00208.x (2001).

11 Oswald, J. C. & Schuster, N. M. Lasmiditan for the treatment of acute migraine: a review and potential role in clinical practice. *J Pain Res* **11**, 2221-2227, doi:10.2147/JPR.S152216 (2018).

12 Rubio-Beltran, E. *et al.* Characterization of binding, functional activity, and contractile responses of the selective 5-HT1F receptor agonist lasmiditan. *Br J Pharmacol* **176**, 4681-4695, doi:10.1111/bph.14832 (2019).
